# Stereoselective chemo-enzymatic oxidation routes for (1R,3E,7E,11S,12S)-3,7,18-dolabellatriene

**DOI:** 10.3389/fmicb.2015.01115

**Published:** 2015-10-13

**Authors:** Christian Görner, Max Hirte, Stephanie Huber, Patrick Schrepfer, Thomas Brück

**Affiliations:** Fachgebiet für Industrielle Biokatalyse, Department für Chemie, Technische Universität MünchenGarching, Germany

**Keywords:** diterpenes, dolabellanes, chemo-enzymatic synthesis, hydroboration, epoxidation

## Abstract

The diterpene (1R,3E,7E,11S,12S)-3,7,18-dolabellatriene from the marine brown alga *Dilophus spiralis* belongs to the dolabellanes natural product family and has antimicrobial activity against multi-drug resistant *Staphylococcus aureus*. Recently, we generated a CotB2 diterpene synthase mutant (W288G), which instead of its native product cyclooctat-9-en-7-ol, generates (1R,3E,7E,11S,12S)-3,7,18-dolabellatriene. *In vivo* CotB2 W288G reconstitution in an *Escherichia coli* based terpene production system, allowed efficient production of this olefinic macrocycle. To diversify the 3,7,18-dolabellatriene bioactivity we evaluated chemical and enzymatic methods for selective oxidation. Epoxidation by acetic peracid, which was formed *in situ* by a lipase catalyzed reaction of acetic acid with H_2_O_2_, provided efficient access to two monooxidized dolabellanes and to a novel di-epoxidated dolabellane species. These compounds could act as synthons en-route to new dolabellanes with diversified bioactivities. Furthermore, we demonstrate the almost quantitative 3,7,18-dolabellatriene conversion into the new, non-natural compound (1R,3E,7E,11S,12S,18R)-dolabella-3,7-diene-20-ol by hydroboration–oxidation with an enantiomeric excess of 94%, for the first time.

## Introduction

**FIGURE 1:**
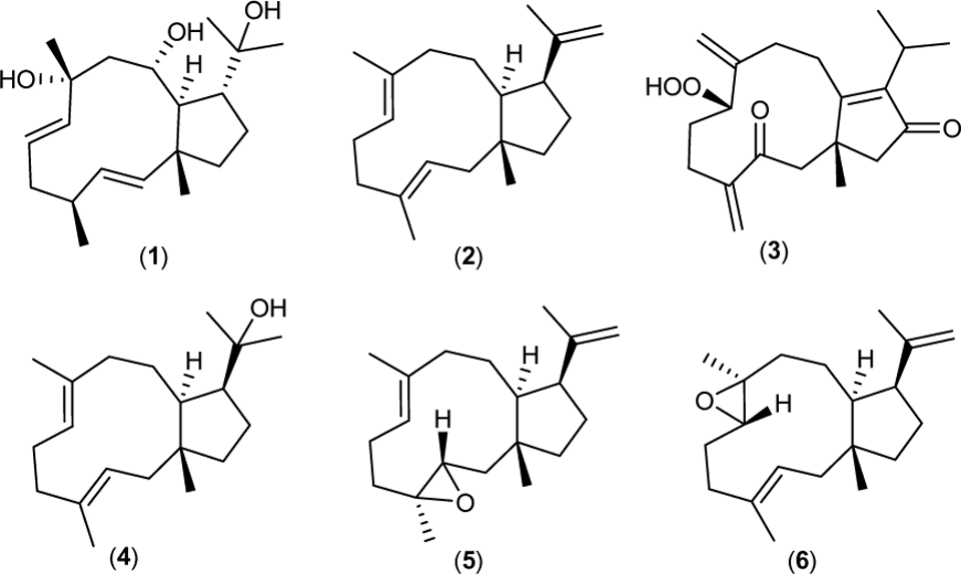
**Bioactive dolabellane structures**.(**1**) from the brown alga *Dictyota pfaffii* inhibits the replication of the Human immunodeficiency virus type-1 (HIV-1). (**2,4,5,6**) from the brown alga *Dilophus spiralis* display antimicrobial activity against *Staphylococcus aureus*. (**3**) isolated from the soft coral *Clavularia inflate* exerts cytotoxic activity against human lung carcinoma cells.

Dolabellane diterpenoids have originally been reported in the herbivor sea hare *Dolabella california* (Ireland and Faulkner, 1977). In later studies marine algae, sponges, and terrestrial plants such as liverworts were found to be rich sources of dolabellane natural products (Cai et al., 2010). All dolabellane diterpenoids share a 5,11-bicyclic skeleton and are frequently functionalized by hydroxyl, epoxide, and glucoside groups. The dolabellane family is known for its broad spectrum of biological activities (see **Figure 1**) encompassing antiviral (**1**) (Cirne-Santos et al., 2008), antibacterial (**2**), (**4**–**6**) (Ioannou et al., 2011), cytotoxic (**3**) (Duh et al., 2001), molluscicidal, and phytotoxic activity (Piattelli et al., 1995).

The efficient total chemical synthesis or biotechnological production process for structurally complex terpenoids such as dolabellanes, arteminisin, or taxol remains challenging (Kusama et al., 2000; Lozama and Prisinzano, 2009; Malik et al., 2011). Total chemical synthesis of functionalized natural products often involves multiple steps, resulting in racemic intermediates, and low product yields. In this respect, the synthetic assembly of olefinic macrocycles containing numerous stereocenters and often display elaborate functionalization patterns, such as glycosylation or addition of aromatic residues, are particularly demanding. Biotechnological routes for stereoselective production of functionalized terpenes are an increasingly attractive alternative to synthetic chemistry but at present remain rather inefficient (Brück et al., 2014). While biosynthetic routes for generation of olefinic terpene macrocycles in heterologous microbial production systems gain significant momentum due to advances in synthetic biotechnology (Leonard et al., 2010), the identification and reconstitution of enzymes that functionalize terpene macrocycles in the desired heterologous production host, remains a bottleneck (Brück et al., 2014). In particular, efficient application of specific P450-type enzyme activities that can hydroxylate aliphatic diterpene macrocycles, en-route to highly functionalized terpenoids, often fail in target-oriented biosynthesis approaches (Zhang et al., 2011).

A strategy to circumvent these obstacles, is the efficient cell based production of terpene macrocycles in suitable *Escherichia coli* or *Saccharomyces cerevisiae* production strains (Engels et al., 2008; Ajikumar et al., 2010; Brück et al., 2014), followed by *in vitro* functionalization utilizing various chemical or enzymatic processes. These semisynthetic routes can provide efficient access to functionalized, bioactive terpenoids, but depend on the availability of the respective terpene synthase and its functional reconstitution in an efficient *in vivo* production system as well as the implementation of selective chemical synthesis steps.

Several chemical strategies have been used in semisynthetic approaches to convert terpenoids to diverse compounds. (Toyooka et al., 1993; Baloglu and Kingston, 1999; Morrison and Hergenrother, 2014; Turconi et al., 2014). However, these chemical strategies conventionally rely on available functionalized precursors, which are derived from isolated natural products or biotechnological production routes that provide specific biosynthetic intermediates. In certain instances the initial hydroxylation of the macrocycle has been accomplished by P450 type oxidoreductases (Le-Huu et al., 2015). By contrast, selective chemical hydroxylation of terpene macrocycles is extremely challenging due to the presence of chemically almost equivalent C-H bonds. Nevertheless, allylic hydroxylation by SeO2-mediated oxidation represent one successful approach. However, this particular reaction suffers from poor selectivity (Katsuyama et al., 2002).

**FIGURE 2:**
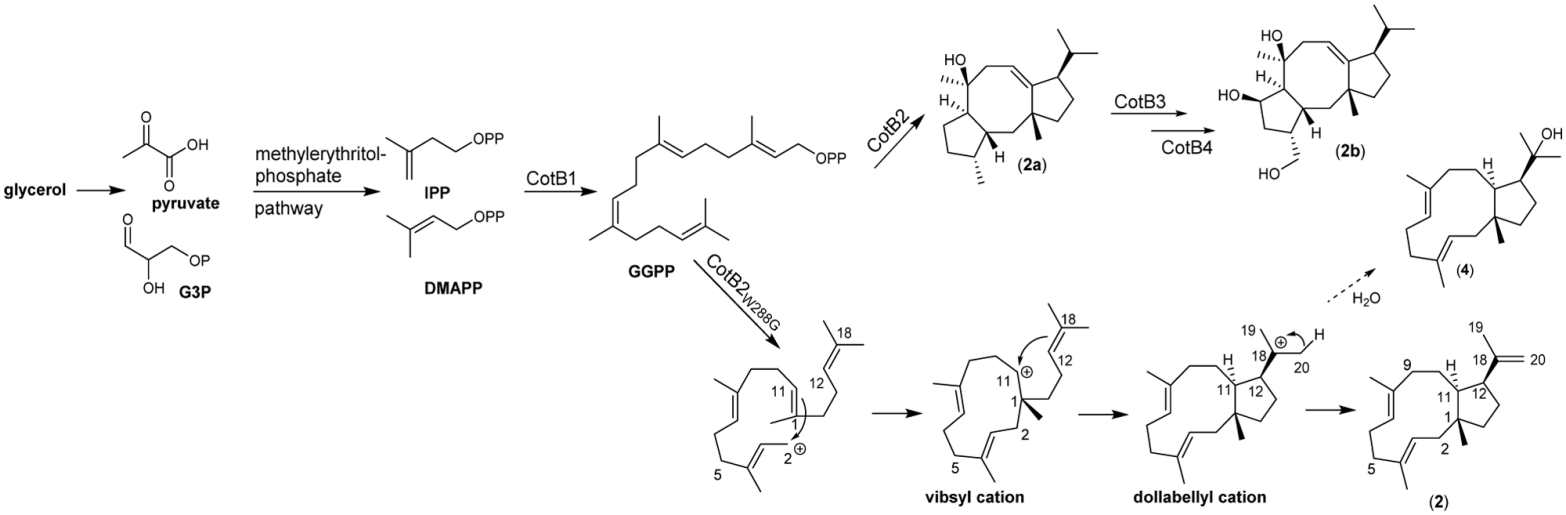
**Biocatalysis and cyclisation mechanism of (1R,3E,7E,11S,12S)-3,7,18-dolabellatriene (**2**)**.

Due to a lack of a native dolabellane synthase the biotechnological production of this diterpene macrocycle and its application in an enzymatic and or a semisynthetic approach has hitherto not been feasible. However, we could previously demonstrate that introduction of the mutation W288G in the diterpene cyclase CotB2, which naturally produces the structurally related fusicoccan cyclooctat-9-en-7-ol (**2b**), instead results in formation of (1R,3E,7E,11S,12S)-3,7,18-dolabellatriene (**2**) (**Figure 2**) (Janke et al., 2014).

The diterpene synthase CotB2 is part of the *Streptomyces melanosporofaciens* derived, four component biosynthetic gene cluster spanning *cotB1* to *cotB4*, which results in formation of cyclooctatin, a lysophospholipase inhibitor (see **Figure 2**; Kim et al., 2009). The reported cyclooctatin biosynthesis pathway encodes a geranylgeranyl diphosphate synthase (CotB1), a diterpene synthase (CotB2) and the class I P450 type hydroxylases CotB3 and CotB4 (**Figure 2**). Interestingly, (**2**), which displays antibacterial activity again multi-drug resistant *Staphylococcus aureus* (MRSA) was first been reported in the brown algae *Dilophus spiralis*. (Ioannou et al., 2011).

The biosynthesis of dolabellane macrocycles starts, like all terpenes, with the stepwise condensation of the universal terpene precursors isopentenyl-diphosphate (IPP) and dimethylallyl diphosphate (DMAPP) yielding the aliphatic diterpene precursor geranylgeranyldiphosphate (C-20). In the subsequent cyclisation reaction by a diterpene synthase, a highly reactive carbocation is created by cleaving of the pyrophosphate group, which propagates the cyclisation through stepwise stereoselective connection of the carbon skeleton. The CotB2 W288G mutation attenuates the cyclisation cascade for the production of cyclooctat-9-en-7-ol by premature proton elimination (Meguro et al., 2015) to yield dolabellatriene (**Figure 2**). In analogy, it can be assumed that a native dolabellane synthase may have a similar reaction mechanism. In this study, we applied both enzymatic and chemical strategies to biotechnological production of dolabellane (2) in order to provide access to a broad spectrum of natural and non-natural dolabellanes.

## Materials and Methods

### General Experimental Procedure

All chemicals were obtained from standard sources at the highest purity grade. NMR spectra were recorded in CDCl_3_ with an Avance-III 500 MHz (Bruker) at 300 K. ^1^H NMR chemical shifts are given in ppm relative to CHCl_3_ (δ = 7.26 ppm). ^13^C NMR chemical shifts are given in ppm relative to CDCl_3_ at δ = 77.16. The 2D experiments (HSQC, COSY, NOESY) were performed using standard Bruker pulse sequences and parameters.

GC-MS analysis of diterpene products from ethyl acetate extractions was done by a Trace GC Ultra with DSQII (Thermo Scientific). One microliter sample was applied by TriPlus AS onto a SGE BPX5 column (30 m, I.D 0.25 mm, Film 0.25 μm). The initial column temperature was 50°C (maintained for 2.5 min). A temperature gradient was applied from 50 to 320°C (10°C/min), followed by 3 min maintenance at 320°C. MS data were recorded at 70 eV (EI), *m/z* (rel. intensity in %) as TIC, total ion current. The recorded *m/z* range was 50–650. Quantification was performed with flame ionization detector (FID) using 1 mg/mL α-humulene (Sigma–Aldrich, Germany) as an internal standard.

### Bacterial Strains, Genes, and Vectors

The *E. coli* strains XL-1 Blue and BL21 (DE3) were used for cloning and diterpene production. All strains and plasmids were obtained from Novagen/Merck Millipore. Genes were synthesized by Life Technologies GmbH featuring the appropriate restriction sites and adjusting codon usage for *E. coli*.

### Plasmids for (1R,3E,7E,11S,12S)-3,7,18-dolabellatriene Production

The 1-deoxy-*D*-xylulose 5-phosphate (DXP) pathway was harbored by the plasmids pCola-Duet-1 and pCDF-Duet-1, while the geranylgeranyl diphosphate synthase (*crtE*) and diterpene synthase *cotB2 W288G* synthase biosynthesis genes were carried by the plasmids pET-Duet-1. To overexpress the DXP pathway in *E. coli* BL21(DE3), genes from *E. coli* encoding for the *1-deoxy-D-xylulose 5-phosphate synthase* (dxs) (GenBank: YP_001461602.1) (Supplementary Figure S13), 1-deoxy-*D*-xylulose 5-phosphate reductoisomerase (*dxr*) (GenBank: NP_414715.1) (Supplementary Figure S14), 2-C-methyl-D-erythriol 4-phosphate cytidyltransferase synthase (*ispD*) (GenBank: NP_417227.1) (Supplementary Figure S15), 2-C-methyl-D-erythritol 2,4-cyclodiphosphate synthase (*ispF*) (GenBank: NP_289295.1) (Supplementary Figure S15), and isopentenyl-diphosphate delta isomerase (*idi*) (GenBank: NP_417365.1) (Supplementary Figure S16) were synthesized. *IspD/ispF* was created as a bi-cistronic operon (Supplementary Figure S15). The native geranylgeranyl diphosphate synthase (*crtE*) (GenBank: M90698.1) sequence was amplified from *Pantoea agglomerans* (ATCC 27155) using standard protocols. Primers used were 5′-AAA CCA TGG CAA TGG CAA CGG TCT GCG CA-3′ and 5′-AAA GAA TTC TTA ACT GAC GGC AGC GAG TTT-3′. The cyclooctat-9-en-7-ol diterpene synthase *cotB2* (GenBank: BAI44338.1) from *Streptomyces melanosporofaciens* MI614-43F2 was synthesized (Supplementary Figure S17). The point mutation W288G in *cotB2* gene for the production of (1R,3E,7E,11S,12S)-3,7,18-dolabellatriene was introduced by the QuikChange site-directed mutagenesis protocol. Primers used were: 5′-CTG ATT TAT GGC AAT TTT GTG GGC ACC ACC TCC AAC AAA CGT TAT AAA AC-3′ and 5′-GTT TTA TAA CGT TTG TTG GAG GTG GTG CCC ACA AAA TTG CCA TAA ATC AG-3′. The synthetic genes and PCR products were introduced into the appropriate plasmids according to **Table 1** by standard cloning techniques.

**Table 1:** Plasmids used to construct the pathway for the production of (1R,3E,7E,11S,12S)-3,7,18-dolabellatriene in *Escherichia coli* BL21 (DE3).

Gene(s)	Vector	Multiple cloning site	Restriction sites
*dxr*	pCola-Duet-1	I	*Nco*I, *EcoR*I
*dxs*	pCola-Duet-1	II	*Nde*I, *Xho*I
*ispD*/*ispF* operon	pCDF-Duet-1	I	*Nco*I, *EcoR*I
*idi*	pCDF-Duet-1	II	*Nde*I, *Xho*I
*crtE*	pET-Duet-1	I	*Nco*I, *EcoR*I
*cotB2*	pET-Duet-1	II	*Nde*I, *Xho*I

### Plasmids Used to Evaluate (1R,3E,7E,11S,12S)-3,7,18-dolabellatriene Oxidation by P450_BM3_

For the *in vitro* and *in vivo* assay of *P450_BM3_* the native gene sequence (Genebank: CP009920.1) was amplified from *Bacillus megaterium* by the following primers: 5′- TAT ACC ATG GCA ATT AAA GAA ATG CCT C-3′ and 5′-ATT AGC GGC CGC TCA CCC AGC CCA CAC G-3′ using standard protocols. The gene was cloned into the vectors pET28a and pACYC-Duet-1 using the restriction sites NcoI and PacI according standard cloning techniques. Mutant version of *P450_BM3_* were created in the vectors pET28a and pACYC-Duet-1, respectively. The mutations F87A and A328 were introduced by the QuikChange site-directed mutagenesis protocol. Primers for the F87A mutation was 5′-GCA GGA GAC GGG TTA GCG ACA AGC TGG ACG CAT G-3′ and 5′-CAT GCG TCC AGC TTG TCG CTA ACC CGT CTC CTG C-3′. For the A328L mutation the following primers were used: 5′-CAT ATA GGG AAA ACG CAG GCA CAG TTG GCC ATA AGC GCA G-3′ and 5′-CTG CGC TTA TGG CCA ACT GTG CCT GCG TTT TCC CTA TAT G-3′.

### Production of (1R,3E,7E,11S,12S)-3,7,18-dolabellatriene

The vectors pCola-Duet-1 (*dxr*, *dxs*), pCDF-Duet-1 (*ispD*/*ispF*, *idi*), and pET-Duet-1 (*crtE*, *cotB2*) were introduced into *E. coli* BL21 (DE3) by standard transformation procedures. The cells were grown in 1 L M9 media supplemented with 1 g/L casamino acids, 30 μg/mL kanamycin, 50 μg/mL streptomycin, 50 μg/mL, and carbenicillin in 5 L baﬄed glass flasks. The culture was inoculated at OD_600_ 0.1 from an overnight culture (8 h cultivation at 37°C, LB media supplemented with 30 μg/mL kanamycin, 50 μg/mL, streptomycin, and 50 μg/mL carbenicillin) and cultivated at 37°C to OD_600_ 0.8. Next, protein expression was started by adding 0.5 mM isopropyl β-D*-*1-thiogalactopyranoside (IPTG). Furthermore, 2% (*w/w*) glycerol and 2.5 g/L hydrophobic beads (Polygoprep 60-50 C18, Macherey-Nagel) were supplemented and cultivation was performed at 28°C for 5 days. After 3 days additional 2% (*w/w*) glycerol were added.

### Extraction and Isolation of (1R,3E,7E,11S,12S)-3,7,18-dolabellatriene

The hydrophobic beads were recovered by filtration, washed with water and subsequently dried. Subsequently, these beads were extracted three times with 200 mL ethyl acetate. The organic phases were combined, dried with MgSO_4_. The solid formed after vacuum evaporation was used in analytical procedures. GC-MS and -FID analysis was carried out with the crude extract, which was dissolved in 1 mL ethyl acetate prior to injection. Purification of (1R,3E,7E,11S,12S)-3,7,18-dolabellatriene was carried out by flash chromatography using hexane on silica (Silica gel 40, Sigma–Aldrich) as solid phase.

### Epoxidation of (1R,3E,7E,11S,12S)-3,7,18-dolabellatriene

15 mg (1R,3E,7E,11S,12S)-3,7,18-dolabellatriene was solved in 10 mL ethyl acetate supplemented with 10 μL acetic acid and 300 mg lipase B resin (lipase acrylic resin from *Candida antarctica*, Sigma–Aldrich) and 5 mM 30% H_2_O_2_ (aq). The mixture was incubated at 25°C for 72 h. For GC-MS and -FID analysis 200 μl aliquots were taken. Samples were washed with 500 μL ddH_2_O and the organic phase was analyzed. After termination of the reaction, the epoxidated dolabellane (**7**) was isolated. To purify the di-epoxidated dolabellane (**7**) a flash chromatography was carried out. Therefore the reaction mixture was dried with MgSO_4_, filtered and evaporated under vacuum to dryness. Purification was carried out by flash chromatography over silica (Silica gel 40, Sigma–Aldrich) utilizing an isocratic 70/30 hexane/ethyl acetate mobile phase.

### Biocatalytical Oxidation of (1R,3E,7E,11S,12S)-3,7,18-dolabellatriene by P450_BM3_

#### *In vitro* Assay

For the *in vitro* assay pET28a(*P450_BM3_*_F87A) and, respectively, pET28a(*P450_BM3_*_F87A/A328L) were introduced into *E. coli* BL21(DE3) by standard transformation methods. From an overnight culture (8 h cultivation at 37°C, LB media supplemented with 30 μg/mL kanamycin) the cells were grown to an OD_600_ 0.5 in 100 mL LB media. Subsequently, 1 mM isopropyl IPTG, 1 mM δ-aminolevulinic acid (ALA), 0.5 mM FeSO_4_ × 7H_2_O and 5 μg/L riboflavin was added to the cultures. Protein expression was done overnight at 25°C. Next, the cells were harvested and centrifuged (10.000*g* for 20 min at 4°C). The cell pellet was resuspended in 5 mL 50 mM kaliumphosphate buffer (pH 7.5). The cells were lysed by sonification using a Sonoplus HD2070 (Bandelin Electronic) performing five repeats on ice (1 min on and 3 min off at 50% power) and subsequently centrifuged (20.000*g* for 20 min at 4°C). For the assay, 100 μL supernatant were added to 400 μL 50 mM kaliumphosphate buffer (pH 7.5) supplemented with 200 μM (1R,3E,7E,11S,12S)-3,7,18-dolabellatriene, which was added in absolute ethanol to a final assay concentration of 0.5% (*v/v*). The reaction was started by adding 100 μM NADPH/H^+^ at 25°C. After 3 h the reaction was stopped by addition of 500 μL ethyl acetate. The reaction mixture was vigorously mixed and the organic phase analyzed by GC-MS.

#### *In vivo* Assay

For the *in vivo* assay the vectors pCola-Duet-1 (*dxr*, *dxs*), pCDF-Duet-1 (*ispD*/*ispF*, *idi*), pET-Duet-1 (*crtE*, *cotB2_W288G_*), and the vector pACYC-Duet-1 (*P450_BM3__F87A / F87A_A328L*) were introduced into *E. coli* Bl21 (DE3) by standard transformation procedures. Cultivation was done in shake flasks and the cells were grown in 400 mL LB medium supplemented with 100 mM HEPES (pH 7.6), 30 μg/mL kanamycin, 50 μg/mL streptomycin, 50 μg/mL carbenicillin, and 34 μg/mL chloramphenicol inoculated from an overnight culture with the same media composition. The cells were grown to an OD_600_ of 0.8 at 37°C. Next, 2% (*v/v*) glycerol, 1 mM δ-aminolevulinic acid (ALA), 1 mM FeSO_4_⋅7H_2_O and 1 mM isopropyl IPTG were added and the cells were cultivated at 25°C for 5 days. (1R,3E,7E,11S,12S)-3,7,18-dolabellatriene and its hydroxylated variants were extracted from cells and supernatant separately after an initial centrifugation step (15 min, 17,500 *g*, 4°C). The cell pellet was washed, suspended in 5 mL water, lysed by sonification using a Sonoplus HD2070 (Bandelin Electronic) performing five repeats on ice (5 min on and 3 min off at 80% power) and subsequently extracted three times with 25 mL ethyl acetate. The supernatant was extracted three times with 200 mL ethyl acetate. Organic phases were combined, dried with MgSO_4_ and evaporated under vacuum to dryness. The crude extract was solved in 1 mL ethyl acetate and analyzed by GC-MS.

### Molecular Docking of (1R,3E,7E,11S,12S)-3,7,18-dolabellatriene in the P450_BM3__F87A and P450_BM3__F87A/A328L Active Side

Molecular Docking was performed using the AutoDock Vina program environment of YASARA structure with the PDB 2X7Y. PDB 2X7Y already contained the F87A mutation. Docking simulation was done with P450BM3 F87A and P450BM3 A87A/A328L. The mutation A328L was introduced in the PDB 2X7Y *in silico*. (1R,3E,7E,11S,12S)-3,7,18-dolabellatriene was docked into a simulation cell (Size: X-size = 16Å, Y-size = 16 Å, Z-size = 16 Å, angles: α = 90°, β = 90°, γ = 90°) around the following four residues Ser 89, Thr 260, Gly 271, and Pro 329. 999 docking runs were performed while all atoms of (1R,3E,7E,11S,12S)-3,7,18-dolabellatriene set as rigid. Cluster analysis were performed in the AutoDock Vina program environment and characterized by binding energy [kcal/mol], dissociation constant [pM], and contacting receptor residues. Results are shown in the Supplementary Material.

### Hydroboration–Oxidation of (1R,3E,7E,11S,12S)-3,7,18-dolabellatriene

Five micro gram of (1R,3E,7E,11S,12S)-3,7,18-dolabellatriene (18.4 μmol, 1 eq) and 3.4 mg 9-BBN-Dimer (13.8 μmol, 1.5 eq) were solved in 1 mL CH_2_Cl_2_. The reaction was stirred at room temperature and the process of the reaction was followed by thin layer chromatography. After (1R,3E,7E,11S,12S)-3,7,18-dolabellatriene was completely converted, the reaction mixture was washed two times with 0.5 mL water. The organic solvent was evaporated under vacuum to dryness and the residue was solved in 500 μL MeOH, 400 μL 2M NaOH (aq), and 100 μL H_2_O_2_ (aq) (30%). The mixture was stirred over night at room temperature. Subsequently, the reaction mixture was extracted three times with 1 mL Et_2_O. The organic phases were combined and evaporated under vacuum to dryness. The crude extract was solved in 1 mL ethyl acetate and analyzed by GC-MS and -FID. Purification was carried out with a silica (Silica 247 gel 40, Sigma–Aldrich) flash chromatography column applying an isocratic 90/10 hexane/ethyl acetate gradient.

## Results and Discussion

### Optimizing Biotechnological Dolabellatriene Production

Recovery of (**2**) from dried algae biomass is limited in yield and supply. Ioannou et al. (2011) were able to isolate 0.15 mg/g from the brown algea *D. spiralis*. Due to the high diversity of dolabellane in this brown algae the isolation of (**2**) also involves extensive purification steps. In our previous research we were able to produce small amouts of (**2**) (1.7 mg/L) by an *E. coli* strain overexpressing the recombinant diterpene biosynthesis pathway with the DXP pathway enzymes isopentenyl diphosphate isomerase (*idi*), 1-deoxyxylulose-5-phosphate synthase (*dxs*), 1-deoxy-D-xylulose-5-phosphate reductoisomerase (*dxr*), geranylgeranyl diphosphate synthase (*crtE*) and the CotB2 W288G diterpene synthase mutant (Janke et al., 2014). More recently, we have achieved the microbial biosynthesis of taxadiene and several other diterpenoids with yields up to 80 mg/L using an *E. coli* strain overexpressing, additional 2-C-methyl-D-erythritol 4-phosphate cytidylyltransferase (*ispD*) and 2-C-methyl-D-erythritol 2,4-cyclodiphosphate synthase (*ispF*) (Schrepfer et al., submitted). In this study we have used this improved diterpene production system to improve our previous reported production yields (Janke et al., 2014). By integration of the CotB2 W288G synthase mutant into this enhanced production strain we could increase the yield for dolabetatriene to 8 mg/L in conventional shake flasks cultures. The dolabellatriene was recovered from eight independent 1 l batches of organic extracts. These extracts contained on average 8 mg/L of the target product. The variations between each batch were minor. This scalable biotechnological production route enables independent access of (**2**), which is a prerequisite for subsequent chemical functionalization studies reported here.

In this study, we devised selective chemical oxidation strategies for (**2**) to generate new dolabellanes with enhanced or diversified bioactivities. Conventionally, complex biocatalytic functionalization of diterpene olefins requires initial oxidative activation of the olefinic macrocycle, which *in vivo* is carried out by highly substrate specific P450-type oxidoreductases. Since there are no biocatalysts known in nature that can functionalize the dolabellatriene macrocycle, we evaluated chemical and enzymatic methodologies for the regio- and stereoselective oxidation of (**2**). The specific challenge in any terpene centered semisynthetic approach is the regio- and stereoselective functionalization of chemically very similar C-H bonds. In nature selectivity is provided by steric demand of amino acid residues in the binding pocket of P450 type enzymes (Brück et al., 2014).

### Epoxidation of Dolabellatriene by Lipase Mediated *in situ* Generated Peracetic Acid

**FIGURE 3:**
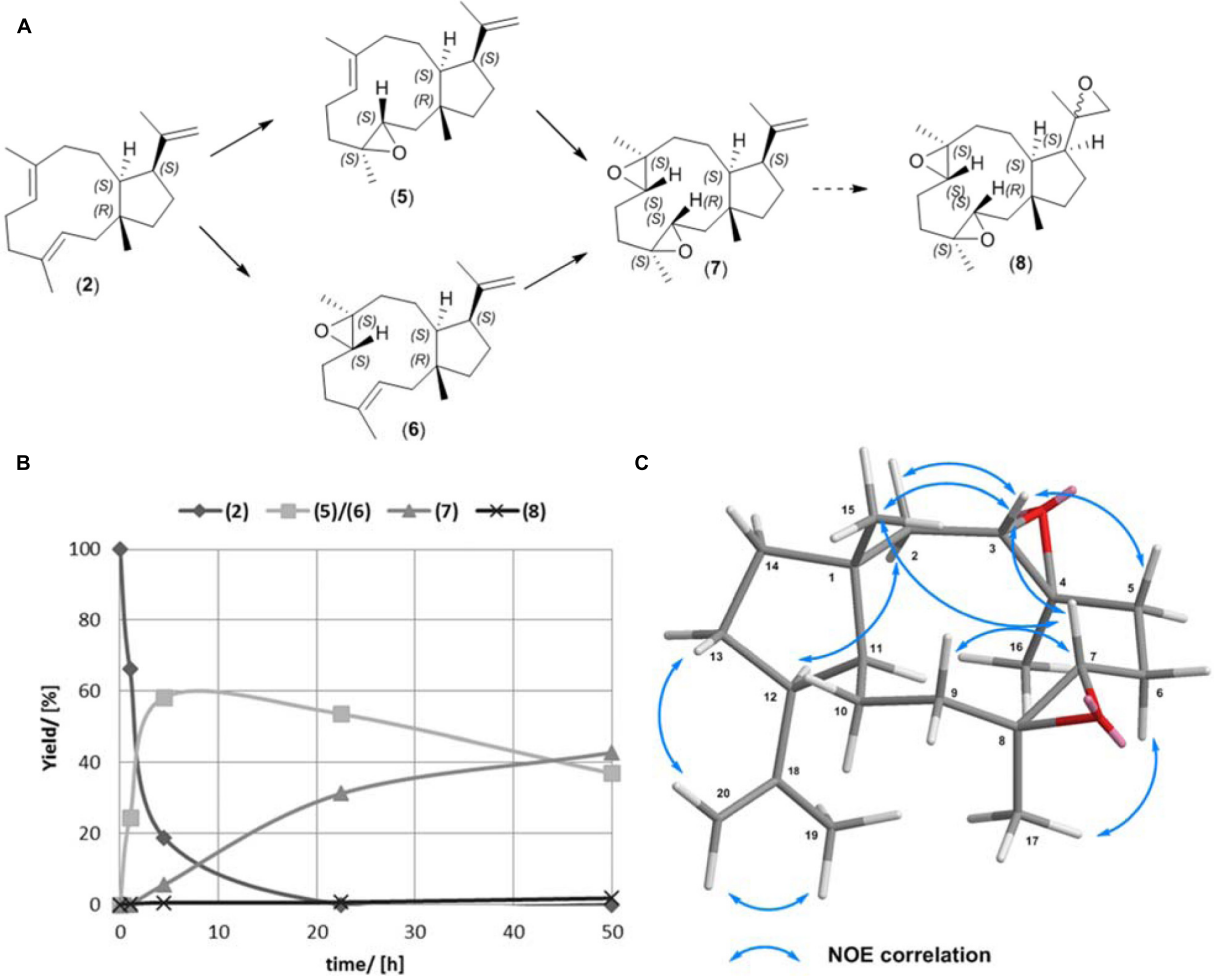
**(A,B) Epoxidation reaction of (1R,3E,7E,11S,12S)-3,7,18-dolabellatriene (**2**). **(C)** Key NOESY correlations of (**7**)**.

In our primary enzymatic based functionalization approach we focused on production of epoxidized variants of (**2**). The structure of (**2**) is in possession of three olefinic bonds. Two are located within the 11 membered ring and show an equal substitution pattern, while the third bond is a terminal alkene with a low degree of substitution. The selective epoxidation of the alkene bonds within the bicyclic carbon skeleton provides the opportunity for the synthesis of (**5**) and (**6**), respectively (**Figure 3**; Ioannou et al., 2011). Since epoxidation by chemical methods is strongly in favor for higher substituted and therefore more reactive dienes, the terminal olefinic bond is discriminated.

Previously, the semisynthetic transformation of (**2**) to the natural, bioactive compounds (**5**), and (**6**) has been demonstrated using *m*-chloroperbenzoic acid, a highly reactive peracid (Ioannou et al., 2011). We evaluated the selectivity of the time resolved epoxidation of 15 mg (**2**) using the less reactive acetic peracid. The reaction was performed in ethyl acetate, containing acetic peracid that was formed *in situ* by reaction of immobilized lipase B (*C. antarctica*) with H_2_O_2_ and catalytic amounts of acetic acid (Da Silva and Nascimento, 2012). The progress of the reaction was monitored by GC-MS analysis (**Figure 3B**). After 4.5 h the highest concentration of a mixture containing equal amounts of (**5**) and (**6**) was detected [58% (5.1 mg, 56% (**5**); 4.1 mg, 44% (**6**)]. At 23 h the yield of (**5**) and (**6**) decreased (53% [4.8 mg, 57% (**5**); 3.7 mg, 43% (**6**))], while the new, di-expoxidated diterpene species (**7**) was formed. The yields of **(5)** and **(6)** observed in this study are similar to those reported for the equivalent reaction in the presence of *m*-chloroperbenzoic acid, which provided 56% [48% (**6**); 52% (**5)]** after 30 min (Ioannou et al., 2011).

The conversion of (**5**) and (**6**) to (**7**) was a rather slow process with a 43% (7.2 mg) yield obtained after 50 h, when the reaction was terminated. In this timeframe (**5**) and (**6**) decreased to 37% [55%, 3.2 mg (**5**); 45%, 2.7 mg (**6**)]. Interestingly, toward the end of the reaction a tri-epoxidated diterpene (**8**) was generated with a limited yield of 1.8% (0.3 mg). Examination of the stereochemistry indicated that the epoxidation of (**2**) was very selective. Since the structure of the non-natural dolabellane (**7**) has not been reported so far, we carried out a comprehensive structural analysis (**Figure 3C**, Supplementary Material). GC-MS and NMR analysis revealed that about 90% of the di-epoxidated (**7**) had the same configuration. Key NOE correlations from NOSEY NMR spectroscopy were observed between H-3/H-7, H-3/H_3_-15, H-2β/H-3, H-7/H-9β, H-7/H_3_-15 and H-2α/H-12 (**Figure 3C**) indicating that the C15-methyl group is at the same side of the molecule as the hydrogens H-3 and H-7 positioned at the epoxy groups. Consequently, the epoxides in (**7**) are likely to display a (S, S) configuration (Supplementary Material). Therefore (**7**) can be described as (1R,3S,4S,7S,8S,11S,12S)-3,4,7,8-diepoxy-dolabella-18-ene. The preference for a certain epoxide conformation can be explained by the higher thermodynamic stability of the (S, S) epoxide compared to the (R, R) counterpart. Nevertheless, during epoxidation the formation of several side products were observed by GC-MS analysis, which may include formation of the (R, R) epoxides. A time resolved monitoring of the lipase mediated dolabellatriene epoxidation allows selective enrichment of either (**5**), (**6**), or (**7**), respectively. Specifically, increased concentrations of (**5**) and (**6**) can be obtained by premature termination of the reaction after 4.5–23 h. Separation of the resulting products (**5**) and (**6**) can be achieved according to previously reported protocols (Ioannou et al., 2011). In contrast to procedures leading to the predominant formation of either **(5)** or **(6)**, enrichment of the di-epoxy diterpene (**7**) requires extended reaction times beyond 30 h. The long reaction times for this side reaction can be reasoned as epoxidation of the terminal olefin group is particularly slow. The resulting products (**5**), (**6**), or (**7**) may be used in a variety of consecutive reactions due to the diversity of epoxide chemistry.

### Stereoselective Hydroxylation of Dolabellatriene by P450_BM3_

In our next enzymatic based functialization approach we focused on functionalization of the less reactive terminal olefinic bond (C-20 C-18) of dolabellatriene. We aimed to transform the olefinic bond into a hydroxyl group, which potentially leads to the production of the more potent dolabellane (**4**) (Ioannou et al., 2011) or related structures.

**FIGURE 4:**
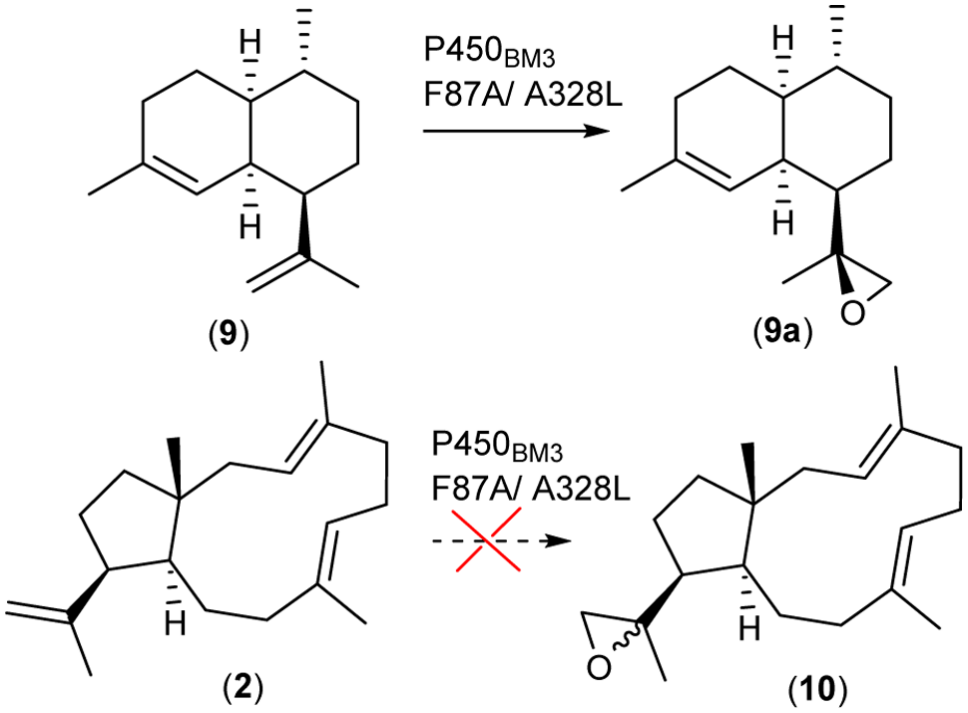
**P450_BM3_ facilitated oxidation of amorpha-4-11-diene**.

Prior to our chemical synthesis approach we elucidated the application of the well-studied hydroxylase P450_BM3_ derived from *B. megaterium*. The enzyme is known to catalyze the hydroxylation of long chain and saturated fatty acids. P450_BM3_ is a fusion protein of a P450 catalytic unit and its corresponding redox system, which displays the highest turnover rate of any currently reported P450 enzyme system (Whitehouse et al., 2012). Consequently, it has been heavily engineered to hydroxylate natural and non-natural substrates, including macrocyclic diterpenoid skeletons (Urlacher and Girhard, 2012). In the case of the sesquiterpenoid macrocycle, amorpha-4-11-diene (**9**) an engineered P450_BM3_ (F87A/A328L) was able to oxidize the terminal olefinic group to artemisinic-11S,12-epoxide (**9a**) (Dietrich et al., 2009). More recently, it has been reported that P450_BM3_ F87A is also capable of oxidizing the bi- hydroxylated diterpenoid β-cembrenediol (Le-Huu et al., 2015). Since epoxides can be used in a variety of consecutive reactions, application of an equivalent approach to generate (**10**) (**Figure 4**) would be very useful. As the epoxidation of (**2**) is more sterically demanding than (**9**), we primarily evaluated the feasibility of this strategy by *in silico* docking experiments (Supplementary Material). The docking experiments did provide an indication that (**2**) may be a substrate for P450_BM3_ (Supplementary Figure S12). Consequently, we generated mutants P450_BM3_ F87A and/or A328L, which have been reported to achieve epoxidation of amorpha-4-11-diene. The P450_BM3_ mutants were recombinantly expressed and tested in an *in vitro* assay supplemented with (**2**). At the end of the assay (3 h) the terpenoids were extracted, concentrated and analyzed by GC/MS. However, none of the assays showed the formation of an oxidized diterpene. The conversion of (**2**) by the P450_BM3_ mutants might be very slow, suffer from inhomogeneous distribution of the substrate and/or result in yields below the limit of detection. Subsequently, we introduced the P450_BM3_ mutants in our *E. coli* based dolabellatriene production system to increase the reaction time and therefore the quantity of potential products. The *E. coli* strain in this *in vivo* assay was cultivated for 5 days followed by a detailed GC/MS analysis. Again we were not able to detect any hydroxylated products, indicating that **(2)** was not a substrate for any of the P450_BM3_ mutants under our investigation. Based on these experimental results, we conclude that our *in silico* docking experiments may have underestimated the steric demands of (**2**) in the enzyme active site. Alternatively it may be argued that an initial introduction of at least one hydroxyl function in the diterpene macrocycle may assist substrate binding and orientation in close proximity to the catalytically active heme iron center. This molecular templating interaction in the active site of P450_BM3_ may explain the successful hydroxylation of β-cembrenediol (Le-Huu et al., 2015). Conversely, for the smaller amorpha-4-11-diene no additional functionalization may be required to mediate binding in close proximity to the catalytically active center. More generally, it can be stated that at present *in silico* calculations do provide any guarantees that particular reactions will take place. However, feedback with experimental data combined with rapid improvements in bioinformatic algorithms may in future facilitate the precise prediction of enzymatic reactions.

Alternative enzyme activities, such as chloroperoxidase, which have been reported to oxidatively activate a variety of terpenes may eventually be capable of utilizing (**2**) as a substrate (Águila et al., 2008; Piantini et al., 2011). We are currently following up with a different strategy, involving high throughput mutagenesis of the class one hydroxylase CotB3, which is naturally involved in formation of cyclooctatin. The rationale behind this approach is the structural similarity of (**2**) with the native CotB3 substrate cyclooctat-9-en-7-ol.

### Stereoselective Hydroxylation of Dolabellatriene via a Hydroboration Strategy

As current enzymatic strategies for hydroxylation of (**2**) were not successful, we evaluated alternative chemical routes for regio- and stereoselective hydroxylation. In order to regio-selectively access the terminal alkene, we evaluated monohydroboration for target orientated hydroxylation. Bulky dialkylboranes are reported to be exceptionally regioselective for terminal alkenes on various substituted dienes (Graham et al., 2011). The resulting trialkylboranes can be used in numerous subsequent reactions to produce alcohols, amines, alkylation, and C-C coupling. Mechanistically, hydroboration is generally an anti-markovnikov reaction, resulting in formation of a primary alcohol after oxidation of the dialkylboranes. Subsequently, we utilized 5 mg of (**2**) as a substrate, which was primarily reacted with 9-borabicyclo[3.3.1]nonane (9-BBN-H) followed by an oxidative step with H_2_O_2_/NaOH. The resulting reaction mixture was analyzed by GC-MS/FID, resulted in detection of two hydroxylation products of (**2**). One of these alcohols was formed in an enantiomeric excess (*ee*) of 94%. The data indicated that the hydroboration reagent preferentially added to one specific side of the terminal olefinic group. The yield of the prominent alcohol (**11**) was determined. After purification the yield of the prominent alcohol (**11**) was 84% (4.5 mg) with respect to the substrate and was subsequently elucidated by 1D and 2D NMR spectroscopy (Supplementary Material). This is the first instance that compound (**11**) is reported.

**FIGURE 5:**
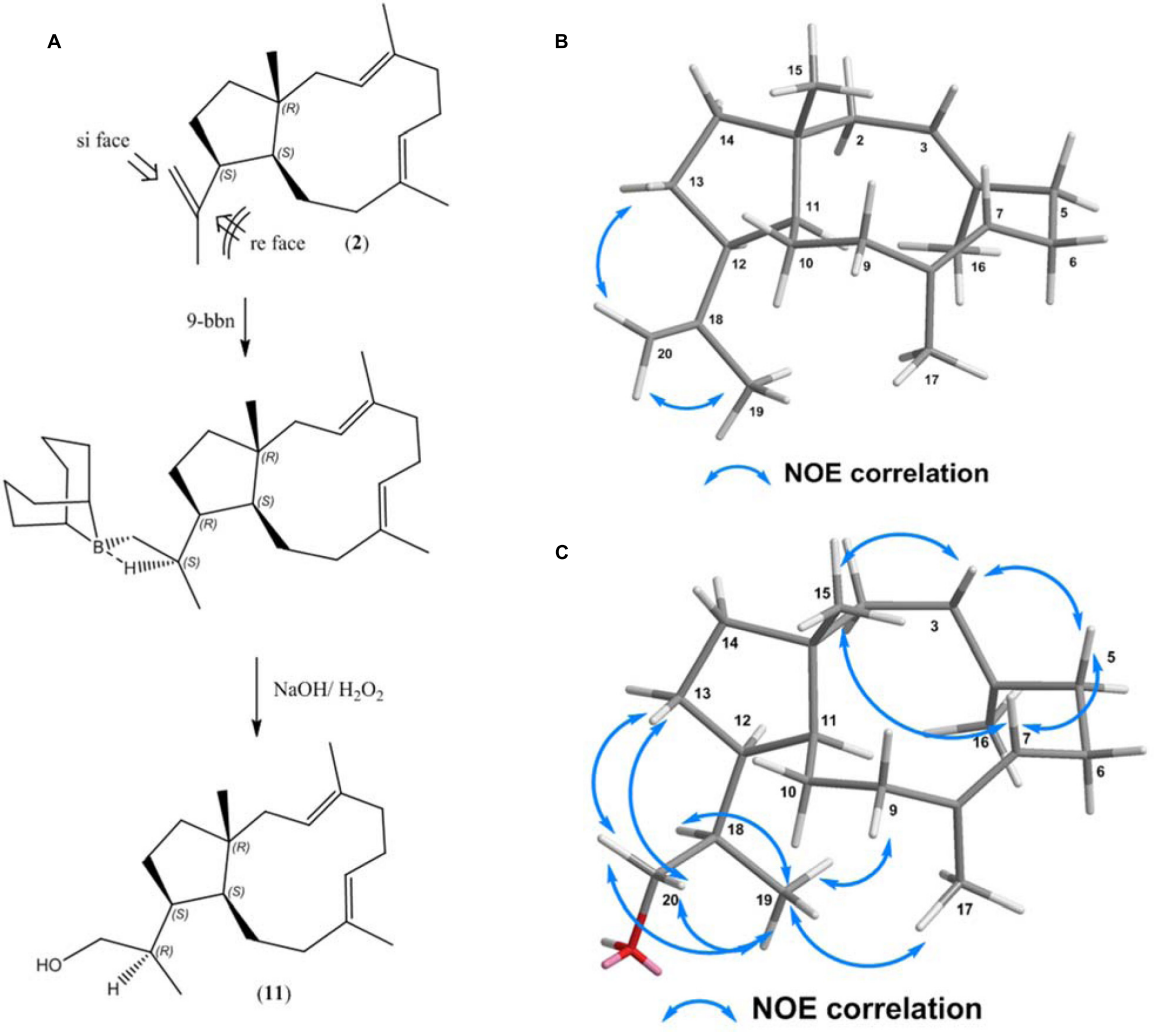
**(A)** Hydroboration–oxidation reaction of (1R,3E,7E,11S,12S)-3,7,18-dolabellatriene (**11**). **(B)** NOESY correlation between H-20α/H_3_-19 and H-20β/H_2_-13 in (**2**) **(C)** Key NOESY correlations for (**11**).

Structure analysis confirmed the stereoselective formation of a primary alcohol (**11**) at the terminal olefinic bond (C-20) (Supplementary Figure S11). The outcome of the hydroboration reaction primarily depends on the position of the terminal alkene group. While in the educt (**2**), the bond between C-12 and C-18 allows free rotation, NOE correlations between H-20α/H_3_-19 and H-20β/H2-13 in (**2**) clearly assign the position of the terminal alkene group to point away from the 11-membered ring of (**2**) (**Figure 5B**). Consequently, mechanistic considerations indicate that the terminal alkene group in (**2**) (see **Figure 5A**) is only accessible to *si face* in the syn addition of 9-BBN, while the *re side* is sterically hindered due to the CH_2_ groups at C9/10 and the C15 methyl group, respectively (**Figure 5A**). The stereochemistry of the newly formed stereo center at C18 was elucidated by NOESY NMR spectroscopy. Key NOE correlations of (**11**) were observed between H_2_-20/H-13, H_2_-20/ H_3_-19, H_2_-20/H-18, H_3_-19/H-9β, H_3_-19/H-18 and are illustrated in **Figure 5B**. In agreement with the observed NOE correlations we suggest the formation of the alcohol (1R,3E,7E,11S,12S,18R)-dolabella-3,7-diene-20-ol (**Figure 5C**). This is the first report of a stereoselective hydroboration of a triene-diterpenoid macrocycle.

## Conclusion

We demonstrated that a sustainable, biotechnological production of the bioactive (1R,3E,7E,11S,12S)-3,7,18-dolabellatriene (**2**) by the mutant diterpene cyclase CotB2 W288G is feasible. This independent route provides access to sufficient material used in subsequent enzymatic and chemical approaches to generate an oxidatively activated macrocycle. Our strategies allowed access of a variety of natural as well as new, non-natural dolabellanes with high regio- and stereo-selectivity. In particular, we could demonstrate that monohydroboration of the differentially substituted terminal triene 3,7,18-dolabellatriene is highly stereoselective. Subsequent oxidation yields the new, non-natural compound (1R,3E,7E,11S,12S,18R)-dolabella-3,7-diene-20-ol (**11**). Consequently, hydroborations may be used in dolabellanes macrocycles to selectively functionalize the terminal alkene group. In a complementary approach we could demonstrate the efficient epoxidation of (**2**) by peracetic acid, which was formed *in situ* using a lipase catalyzed conversion of acetic acid and H_2_O_2_, resulting in products (**5**), (**6**), (**7**), (**8**). By temporal monitoring of the resulting reaction mixture it is possible to selectively enrich these products. In this study we could demonstrate that combining biotechnological, chemo-enzymatic, and chemical reaction cascades for the efficient and scalable production of oxidatively activated diterpene macrocycles. The resulting reaction products may inherently display improved bioactivity compared to their parent compound. Alternatively, these chemically activated compounds may serve as synthons for more elaborate synthesis toward highly functionalized natural and non-natural dolabellanes.

## Conflict of Interest Statement

The authors declare that the research was conducted in the absence of any commercial or financial relationships that could be construed as a potential conflict of interest.

## References

[B1] Águila S. Vazquez-Duhalt R. Tinoco R. Rivera M. Pecchi G. Alderete J. B. (2008). Stereoselective oxidation of R-(+)-limonene by chloroperoxidase from *Caldariomyces fumago*. *Green Chem.* 10 647–653. 10.1039/b719992a

[B2] Ajikumar P. K. Xiao W.-H. Tyo K. E. J. Wang Y. Simeon F. Leonard E. (2010). Isoprenoid pathway optimization for Taxol precursor overproduction in *Escherichia coli*. *Science* 330 70–74. 10.1126/science.1191652 20929806 PMC3034138

[B3] Baloglu E. Kingston D. G. (1999). A new semisynthesis of paclitaxel from baccatin III. *J. Nat. Prod.* 62 1068–1071. 10.1021/np990040k 10425147

[B4] Brück T. Kourist R. Loll B. (2014). Production of macrocyclic sesqui- and diterpenes in heterologous microbial hosts: a systems approach to harness nature’s molecular diversity. *ChemCatChem* 6 1142–1165. 10.1002/cctc.201300733

[B5] Cai X.-H. Wang Y.-Y. Zhao P.-J. Li Y. Luo X.-D. (2010). Dolabellane diterpenoids from *Aglaia odorata*. *Phytochemistry* 71 1020–1024. 10.1016/j.phytochem.2010.03.005 20338601

[B6] Cirne-Santos C. C. Souza T. M. L. Teixeira V. L. Fontes C. F. L. Rebello M. A. Castello-Branco L. R. R. (2008). The dolabellane diterpene Dolabelladienetriol is a typical noncompetitive inhibitor of HIV-1 reverse transcriptase enzyme. *Antiviral Res.* 77 64–71. 10.1016/j.antiviral.2007.08.006 17888523

[B7] Da Silva J. M. R. Nascimento M. da G. (2012). Chemoenzymatic epoxidation of citronellol catalyzed by lipases. *Process Biochem.* 47 517–522. 10.1016/j.procbio.2011.12.019

[B8] Dietrich J. A. Yoshikuni Y. Fisher K. J. Woolard F. X. Ockey D. McPhee D. J. (2009). A novel semi-biosynthetic route for artemisinin production using engineered substrate-promiscuous P450(BM3). *ACS Chem. Biol.* 4 261–267. 10.1021/cb900006h 19271725

[B9] Duh C.-Y. Chia M.-C. Wang S.-K. Chen H.-J. El-Gamal A. A. H. Dai C.-F. (2001). Cytotoxic dolabellane diterpenes from the formosan soft coral *Clavularia inflata*. *J. Nat. Prod.* 64 1028–1031. 10.1021/np010106n 11520220

[B10] Engels B. Dahm P. Jennewein S. (2008). Metabolic engineering of taxadiene biosynthesis in yeast as a first step towards Taxol (Paclitaxel) production. *Metab. Eng.* 10 201–206. 10.1016/j.ymben.2008.03.001 18485776

[B11] Graham T. J. A. Poole T. H. Reese C. N. Goess B. C. (2011). Regioselective Semihydrogenation of Dienes. *J. Org. Chem.* 76 4132–4138. 21520957 10.1021/jo200262r

[B12] Ioannou E. Quesada A. Rahman M. M. Gibbons S. Vagias C. Roussis V. (2011). Dolabellanes with antibacterial activity from the brown alga *Dilophus spiralis*. *J. Nat. Prod.* 74 213–222. 10.1021/np1006586 21190330

[B13] Ireland C. Faulkner D. J. (1977). Diterpenes from *Dolabella californica*. *J. Org. Chem.* 42 3157–3162. 10.1021/jo00439a010 894397

[B14] Janke R. Görner C. Hirte M. Brück T. Loll B. (2014). The first structure of a bacterial diterpene cyclase: CotB2. *Acta Crystallogr. D. Biol. Crystallogr.* 70 1528–1537. 10.1107/S1399004714005513 24914964

[B15] Katsuyama I. Fahmy H. Zjawiony J. K. Khalifa S. I. Kilada R. W. Konoshima T. (2002). Semisynthesis of new sarcophine derivatives with chemopreventive activity. *J. Nat. Prod.* 65 1809–1814. 10.1021/np020221d 12502319

[B16] Kim S.-Y. Zhao P. Igarashi M. Sawa R. Tomita T. Nishiyama M. (2009). Cloning and heterologous expression of the cyclooctatin biosynthetic gene cluster afford a diterpene cyclase and two p450 hydroxylases. *Chem. Biol.* 16 736–743. 10.1016/j.chembiol.2009.006.007 19635410

[B17] Kusama H. Hara R. Kawahara S. Nishimori T. Kashima H. Nakamura N. (2000). Enantioselective total synthesis of (-)-Taxol. *J. Am. Chem. Soc.* 122 3811–3820. 10.1021/ja9939439

[B18] Le-Huu P. Heidt T. Claasen B. Laschat S. Urlacher V. B. (2015). Chemo-. Regio-, and Stereoselective Oxidation of the Monocyclic Diterpenoid β-Cembrenediol by P450 BM3. *ACS Catal.* 5 1772–1780. 10.1021/cs5020404

[B19] Leonard E. Ajikumar P. K. Thayer K. Xiao W.-H. Mo J. D. Tidor B. (2010). Combining metabolic and protein engineering of a terpenoid biosynthetic pathway for overproduction and selectivity control. *Proc. Natl. Acad. Sci. U.S.A.* 107 13654–13659. 10.1073/pnas.1006138107 20643967 PMC2922259

[B20] Lozama A. Prisinzano T. E. (2009). Chemical methods for the synthesis and modification of neoclerodane diterpenes. *Bioorg. Med. Chem. Lett.* 19 5490–5495. 10.1016/j.bmcl.2009.07.069 19679471 PMC2977960

[B21] Malik S. Cusidó R. M. Mirjalili M. H. Moyano E. Palazón J. Bonfill M. (2011). Production of the anticancer drug taxol in *Taxus baccata* suspension cultures: a review. *Process Biochem.* 46 23–34. 10.1016/j.procbio.2010.09.004

[B22] Meguro A. Motoyoshi Y. Teramoto K. Ueda S. Totsuka Y. Ando Y. (2015). An unusual terpene cyclization mechanism involving a carbon-carbon bond rearrangement. *Angew. Chem Int. Ed. Engl.* 127 4427–4430. 10.1002/ange.201411923 25689152

[B23] Morrison K. C. Hergenrother P. J. (2014). Natural products as starting points for the synthesis of complex and diverse compounds. *Nat. Prod. Rep.* 31 6–14. 10.1039/c3np70063a 24219884

[B24] Piantini U. Schrader J. Wawrzun A. Wüst M. (2011). A biocatalytic route towards rose oxide using chloroperoxidase. *Food Chem.* 129 1025–1029. 10.1016/j.foodchem.2011.05.068 25212332

[B25] Piattelli M. Tringali C. Neri P. Rocco C. (1995). Stereochemistry and conformation of dolabellane diterpenes: an NMR and molecular mechanics study. *J. Nat. Prod.* 58 697–704. 10.1021/np50119a007

[B26] Toyooka N. Nishino A. Momose T. (1993). Ring differentiation of the trans-decahydronaphthalene system via chemo-enzymatic dissymmetrization of its σ-symmetric glycol: synthesis of a highly functionalized chiral building block for the terpene synthesis. *Tetrahedron Lett.* 34 4539–4540. 10.1016/0040-4039(93)88079-X

[B27] Turconi J. Griolet F. Guevel R. Oddon G. Villa R. Geatti A. (2014). Semisynthetic artemisinin, the chemical path to industrial production. *Org. Process Res. Dev.* 18 417–422. 10.1021/op4003196

[B28] Urlacher V. B. Girhard M. (2012). Cytochrome P450 monooxygenases: an update on perspectives for synthetic application. *Trends Biotechnol.* 30 26–36. 10.1016/j.tibtech.2011.06.012 21782265

[B29] Whitehouse C. J. C. Bell S. G. Wong L.-L. (2012). P450(BM3) (CYP102A1): connecting the dots. *Chem. Soc. Rev.* 41 1218–1260. 10.1039/c1cs15192d 22008827

[B30] Zhang K. El Damaty S. Fasan R. (2011). P450 fingerprinting method for rapid discovery of terpene hydroxylating P450 catalysts with diversified regioselectivity. *J. Am. Chem. Soc.* 133 3242–3245. 10.1021/ja109590h 21341707

