# Supplementary material for: Stereoselective chemo-enzymatic oxidation routes for (1R,3E,7E,11S,12S)-3,7,18-dolabellatriene
Source: Front Microbiol. 2015 Oct 13;6:1115. doi: 10.3389/fmicb.2015.01115 (PMC4602142; doi:10.3389/fmicb.2015.01115)
Supplement: Supplementary file 1 [file Data_Sheet_1.PDF]

Supplementary Material

Stereoselective chemo-enzymatic oxidation routes for (1R,3E,7E,11S,12S)-3,7,18-dolabellatriene

Christian Goerner[1], Max Hirte[1], Stephanie Huber[1], Patrick Schrepfer[1] and Thomas Brück[1]*

*** Correspondence:** Thomas Brück, [1]Fachgebiet für Industrielle Biokatalyse, Department für Chemie, Technische Universität München, Lichtenberg Str.4 85748 Garching, Germany brueck@tum.de

1. NMR spectral analysis

Supplementary Table 1. ^{13}C Spectral data of (7)

# Position	δ_C (ppm)	Functional Group	# Position	δ_C (ppm)	Functional Group
1	43.63	C	11	42.47	CH
2	43.26	CH_2	12	51.41	CH
3	63.62	CH	13	27.24	CH_2
4	61.21	C	14	41.84	CH_2
5	37.62	CH_2	15	24.69	CH_3
6	23.71	CH_2	16	16.37	CH_3
7	64.73	CH	17	17.36	CH_3
8	61.44	C	18	145.47	C
9	36.53	CH_2	19	24.12	CH_3
10	22.31	CH_2	20	112.57	CH_2

Supplementary Table 2. [1]H Spectral data of (7)

# Position	δ_H *(ppm), J* (Hz)	# Position	δ_H *(ppm), J* (Hz)
1		11	1.81 (m, 1H)
2	1.52 (m, 1H)<br>1.83 (m, 1H)	12	2.67 (m, 1H)
3	2.96 (dd, *J* = 2.75, 11.17, 1H)	13	1.58 (m, 1H)<br>1.71 (m, 1H)
4		14	1.47 (m, 1H)<br>1.61 (m, 1H)
5	1.33 (m, 1H)<br>2.26 (m, 1H)	15	1.24 (s, 3H)
6	1.64 (m, 1H)<br>1.97 (m, 1H)	16	1.32 (s, 3H)
7	2.77 (d, *J* = 8.95, 1H)	17	1.27 (s, 3H)
8		18	
9	1.35 (m, 1H)<br>2.02 (m, 1H)	19	1.75 (s, 3H)
10	1.38 (m, 1H)<br>1.46 (m, 1H)	20	β 4.71 (s, 1H)<br>α 4.94 (s, 1H)

Supplementary Figure 1. Key NOE correlations of (7)

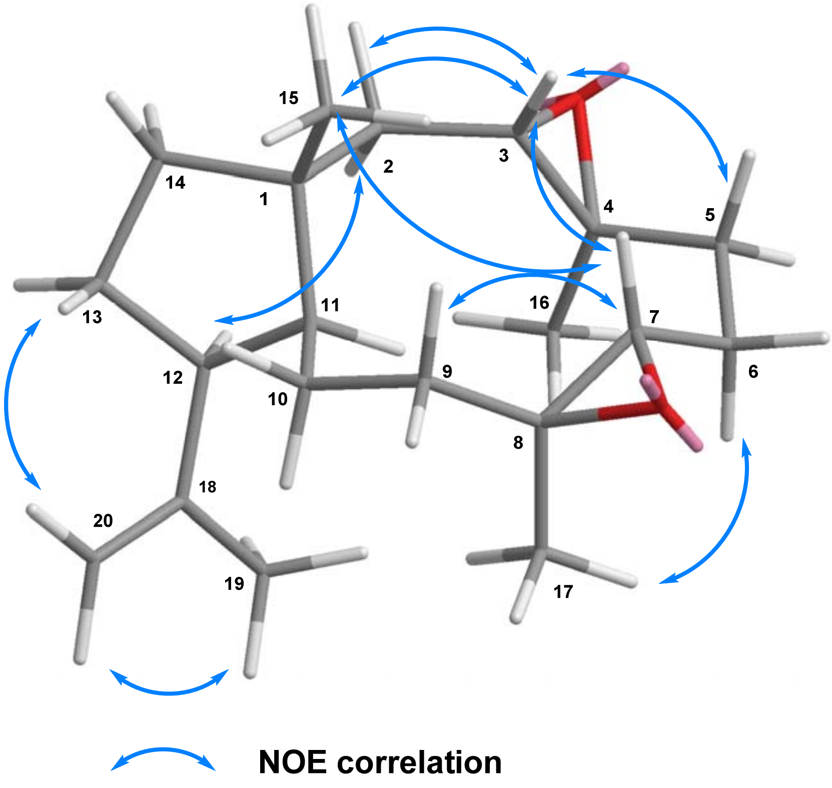

Supplementary Figure 2. ^{1}H NMR (500 MHz, $CDCl_3$) of (7)

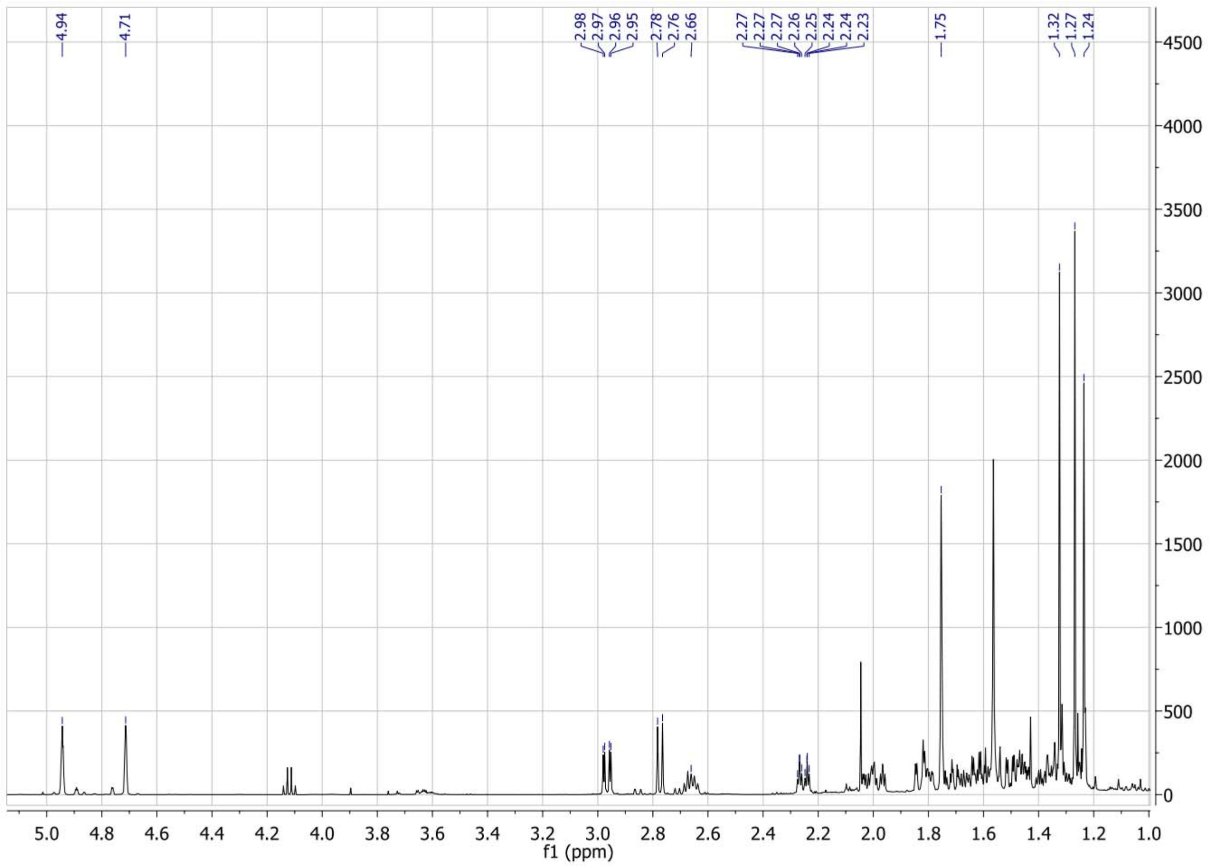

Supplementary Figure 3. ^{13}C NMR (125 MHz, $CDCl_3$) of (7)

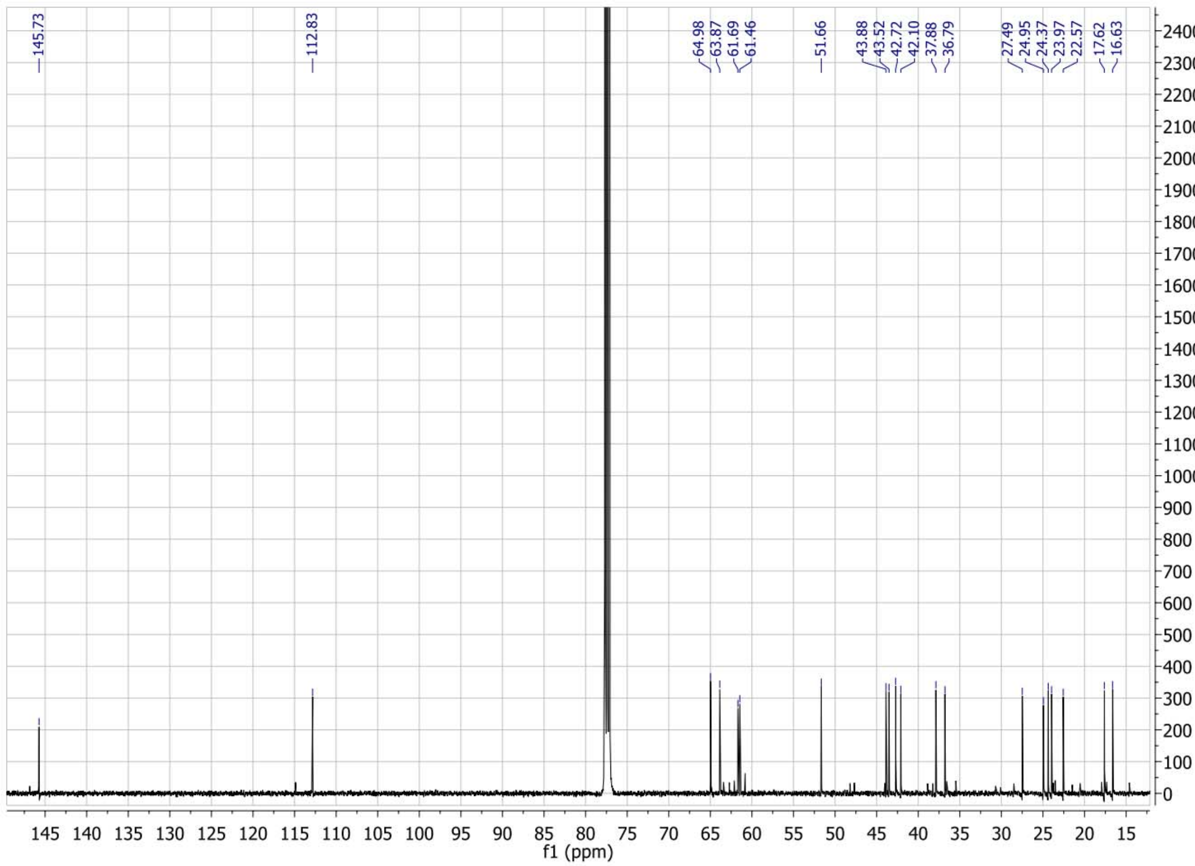

Supplementary Table 3. ^{13}C Spectral data of (11)

# Position	δ_C (ppm)	Functional Group	# Position	δ_C (ppm)	Functional Group
1	46.59	C	11	41.05	CH
2	43.25	CH_2	12	47.33	CH
3	126.19	CH	13	27.77	CH_2
4	133.52	C	14	42.42	CH_2
5	39.96	CH_2	15	25.1	CH_3
6	24.56	CH_2	16	15.89	CH_3
7	128.06	CH	17	16.83	CH_3
8	134.59	C	18	35.45	CH
9	38.08	CH_2	19	17.16	CH_3
10	22.94	CH_2	20	67.83	CH_2

Supplementary Table 4. ^{1}H Spectral data of (11)

# Position	δ_H *(ppm)*, *J* (Hz)	# Position	δ_H *(ppm)*, *J* (Hz)
1		11	1.71 (m, 1H)
2	1.65 (m, 1H)<br>2.20 (m, 1H)	12	1.80 (m, 1H)
3	5.15 (dd, *J* = 11.5, 4.6, 1H)	13	1.34 (m, 1H)<br>1.58 (m, 1H)
4		14	1.42 (m, 2H)
5	2.08 (m, 1H)<br>2.21 (m, 1H)	15	1.08 (s, 3H)
6	2.08 (m, 1H)<br>2.29 (m, 1H)	16	1.50 (s, 3H)
7	4.89 (m, 1H)	17	1.58 (s, 3H)
8		18	1.64 (m, 1H)
9	1.97 (m, 1H)<br>2.24 (m, 1H)	19	0.98 (d, *J* = 6.5, 3H)
10	1.27 (m, 1H)<br>1.46 (m, 1H)	20	3.43 (t, *J* = 8.8 1H)<br>3.68 (d, *J* = 10.4, 1H)

Supplementary Figure 4. ^{1}H NMR (500 MHz, $CDCl_3$) of (11)

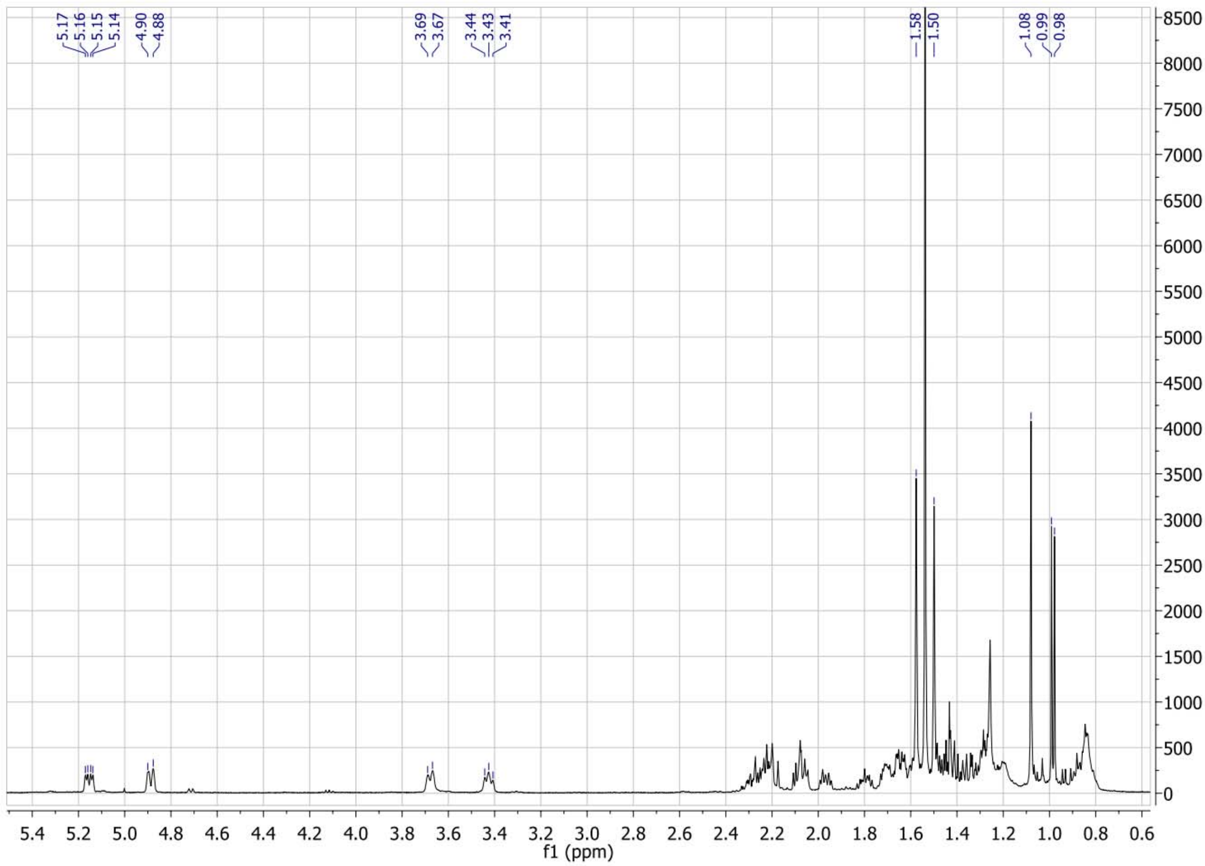

Supplementary Figure 5. ^{13}C NMR (125 MHz, $CDCl_3$) of (11)

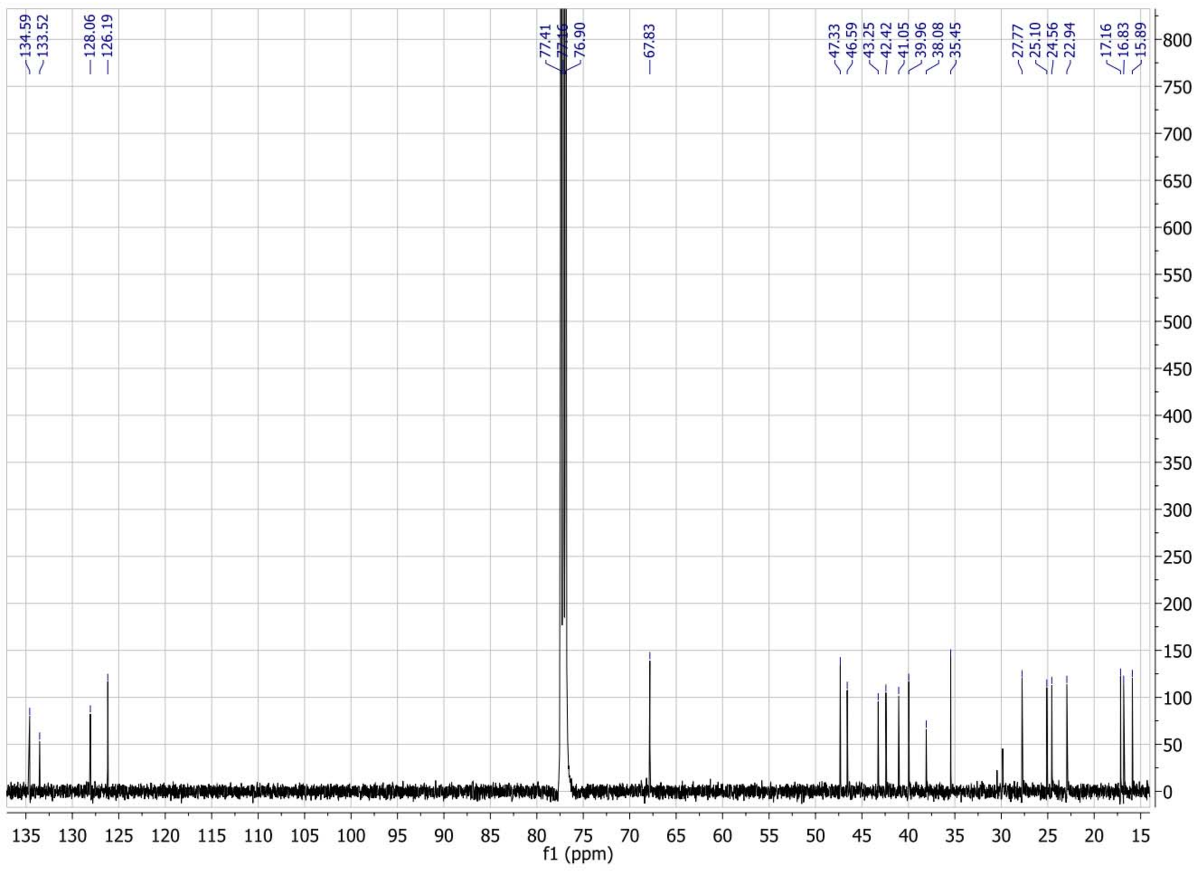

Supplementary Figure 6. Key NOE correlations of (11)

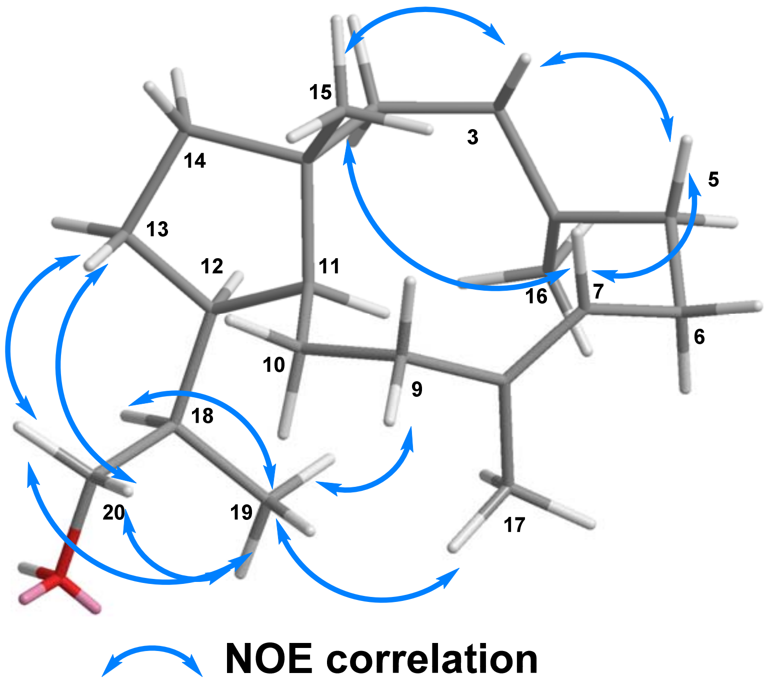

2. Mass spectrometry analysis

Supplementary Figure 7. Mass spectrum of (5), recorded on a Trace GC Ultra with DSQII (Thermo Scientific), m/z was analyzed from [50-650], m/z $C_{20}H_{35}O$ calculated 388.24.

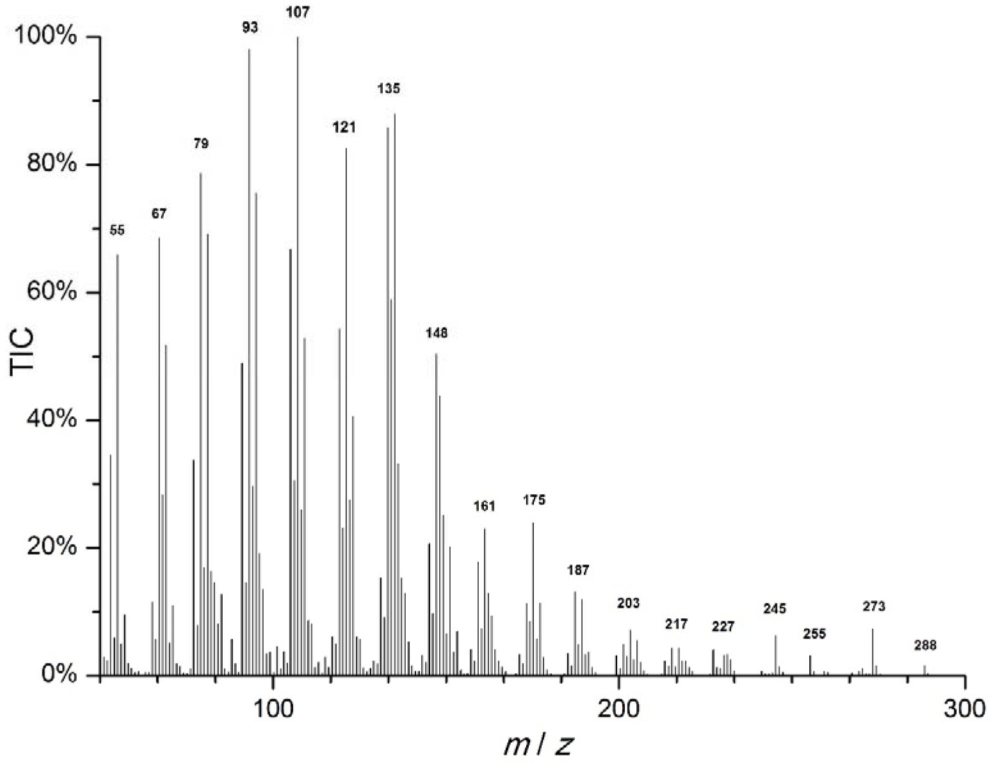

Supplementary Figure 8. Mass spectrum of (6), recorded on a Trace GC Ultra with DSQII (Thermo Scientific), m/z was analyzed from [50-650], m/z $C_{20}H_{35}O$ calculated 388.24.

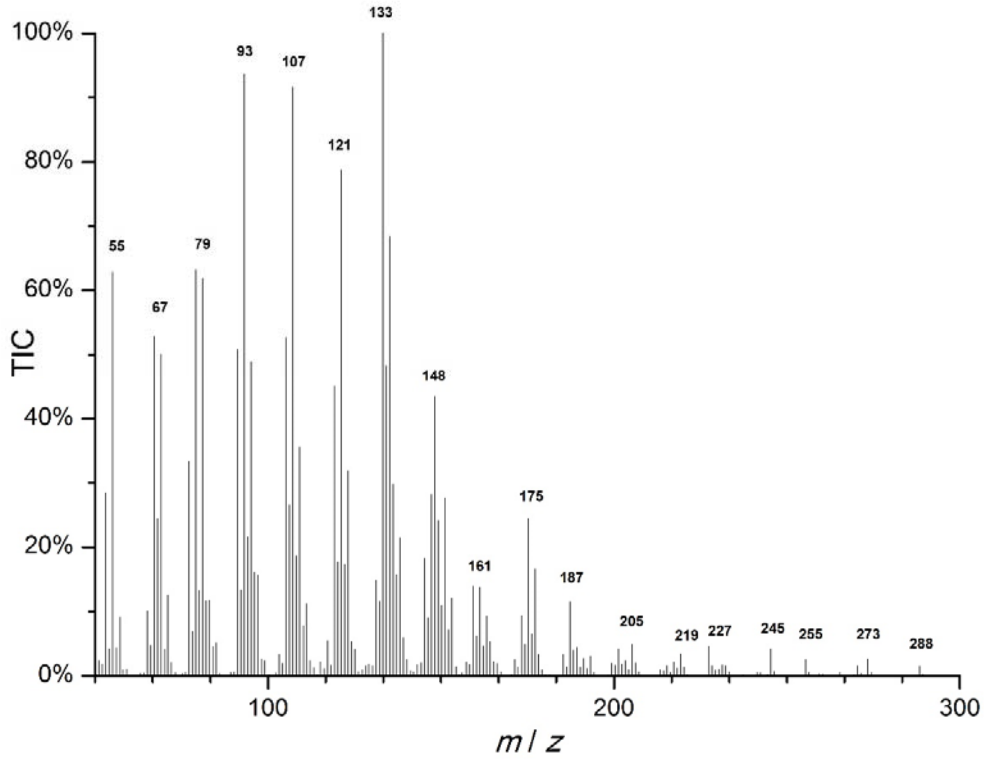

Supplementary Figure 9. Mass spectrum of (7), recorded on a Trace GC Ultra with DSQII (Thermo Scientific), m/z was analyzed from [50-650], m/z $C_{20}H_{35}O$ calculated 304.24.

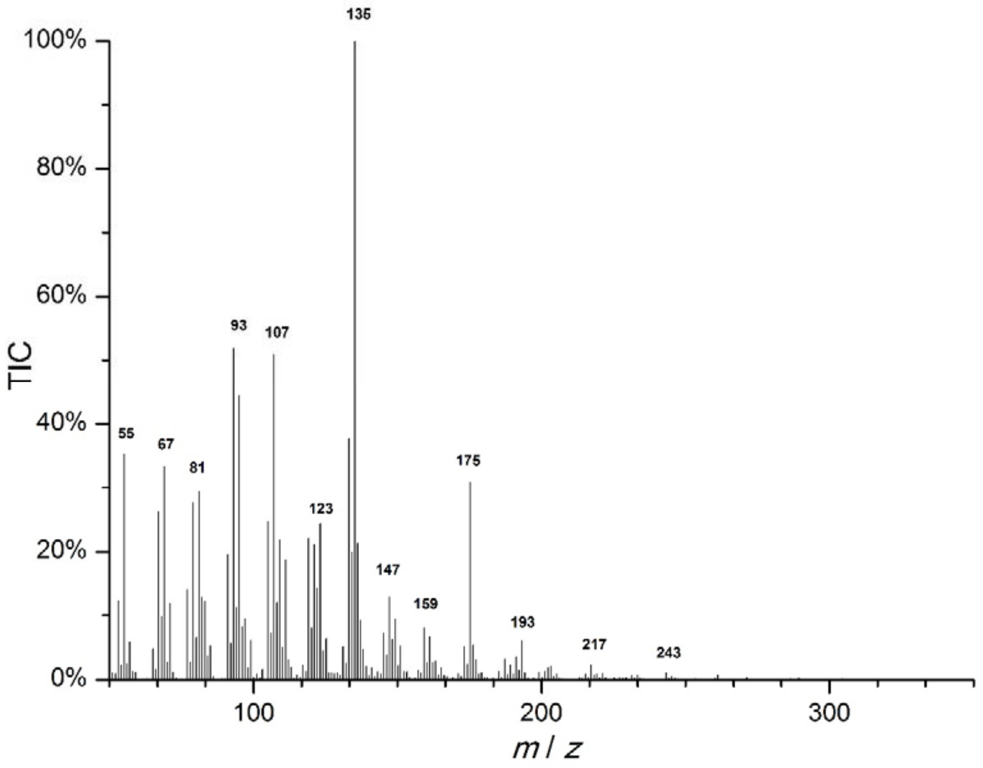

Supplementary Figure 10. Mass spectrum of (8), recorded on a Trace GC Ultra with DSQII (Thermo Scientific), m/z was analyzed from [50-650], m/z $C_{20}H_{35}O$ calculated 320.23.

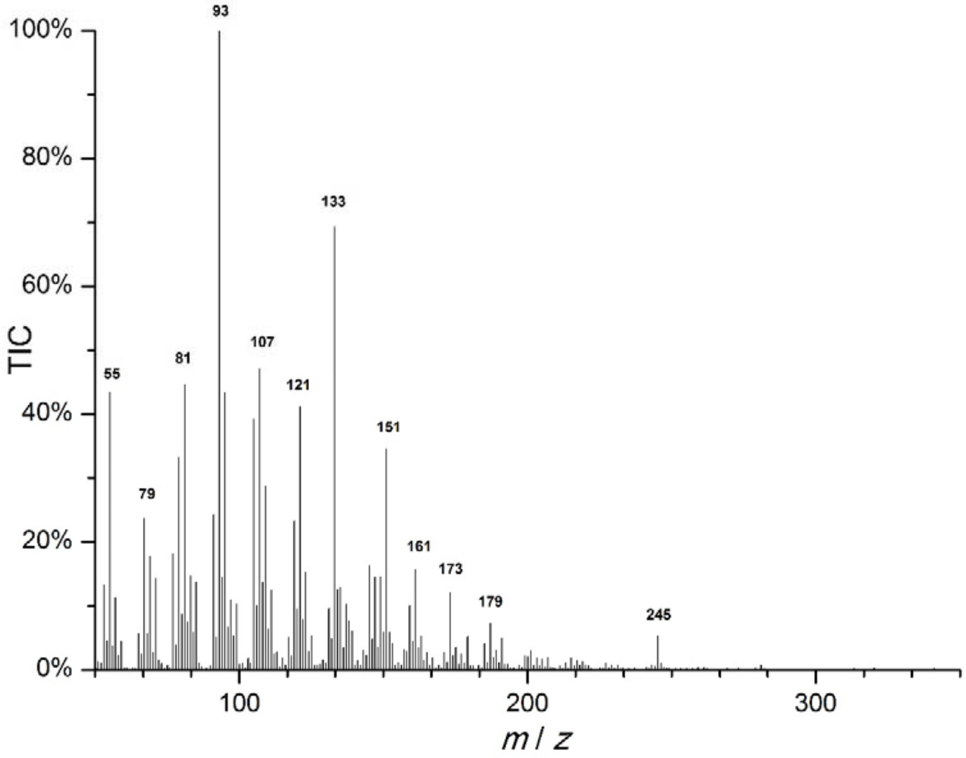

Supplementary Figure 11. Mass spectrum of (11), recorded on a Trace GC Ultra with DSQII (Thermo Scientific), m/z was analyzed from [50-650], m/z $C_{20}H_{35}O$ calculated 290.26.

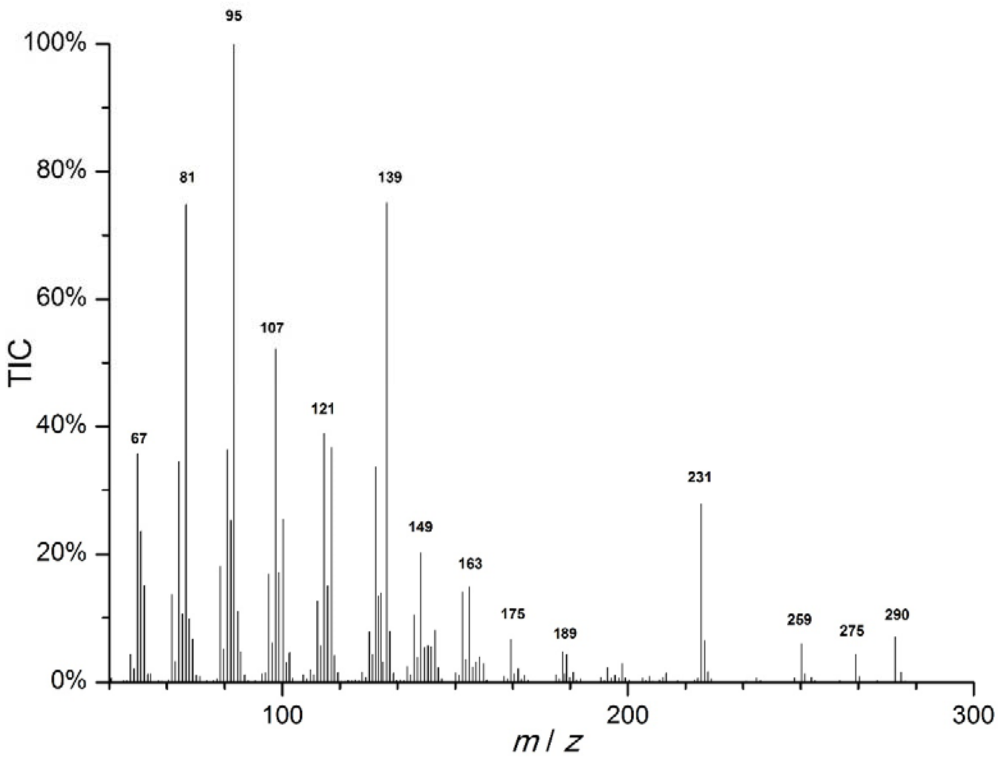

3. Bioinformatic

Docking results of $P450_{BM3}$ F87A with the substrate (1R,3E,7E,11S,12S)-3,7,18-dolabellatriene

After clustering the 999 runs, the following 2 distinct complex conformations were found:

[They all differ by at least 2.0 A heavy atom RMSD]

Clu |Bind.energy[kcal/mol]|Dissoc. constant [pM]| Contacting receptor residues

001 | 000008.8850 | 00000000307049.6875 | LEU A 75 VAL A 78 ALA A 87 THR A 88 LEU A 181 THR A 260 ILE A 263 ALA A 264 GLU A 267 THR A 268 ALA A 328 PRO A 329 ALA A 330 LEU A 437 THR A 438 HEM A 1456

002 | 000008.1180 | 00000001120539.3750 | LYS A 69 SER A 72 ALA A 74 LEU A 75 VAL A 78 ALA A 87 THR A 88 LEU A 181 ALA A 264 THR A 268 ALA A 328 PRO A 329 ALA A 330 PHE A 331 SER A 332 MET A 354 LEU A 437 THR A 438 HEM A 1456

Average and standard deviation:

Clu |Members|Bind.energy spread [kcal/mol]

001 | 007 | 000008.2020+-000000.4795

002 | 008 | 000007.8925+-000000.3807

Docking results of $P450_{BM3}$ F87A/A328L with the substrate (1R,3E,7E,11S,12S)-3,7,18-dolabellatriene

After clustering the 999 runs, the following 2 distinct complex conformations were found:

[They all differ by at least 2.0 A heavy atom RMSD]

Clu |Bind.energy[kcal/mol]|Dissoc. constant [pM]| Contacting receptor residues

001 | 000007.5920 | 00000002722640.0000 | LYS A 69 LEU A 75 VAL A 78 PHE A 81 ALA A 82 ALA A 87 THR A 88 LEU A 181 ILE A 259 THR A 260 ILE A 263 ALA A 264 GLU A 267 THR A 268 LEU A 328 LEU A 437 THR A 438 HEM A 1456

002 | 000005.7350 | 00000062544520.0000 | LYS A 69 LEU A 75 VAL A 78 PHE A 81 ALA A 82 ALA A 87 THR A 88 LEU A 181 THR A 260 ILE A 263 ALA A 264 GLU A 267 THR A 268 LEU A 328 LEU A 437 THR A 438 HEM A 1456

Average and standard deviation]:

Clu |Members|Bind.energy spread [kcal/mol]

001 | 008 | 000005.5711+-000001.2051

002 | 008 | 000005.7521+-000000.8603

Supplementary Figure 12. Modelled $P450_{BM3}$ F87A (green) contains prosthetic heme group (gray) and docked (1R,3E,7E,11S,12S)-3,7,18-dolabellatriene (magenta). The heme group contains the iron cation (orange). (1R,3E,7E,11S,12S)-3,7,18-dolabellatriene is shown in the conformation comprising the lowest binding energy derived from cluster I.

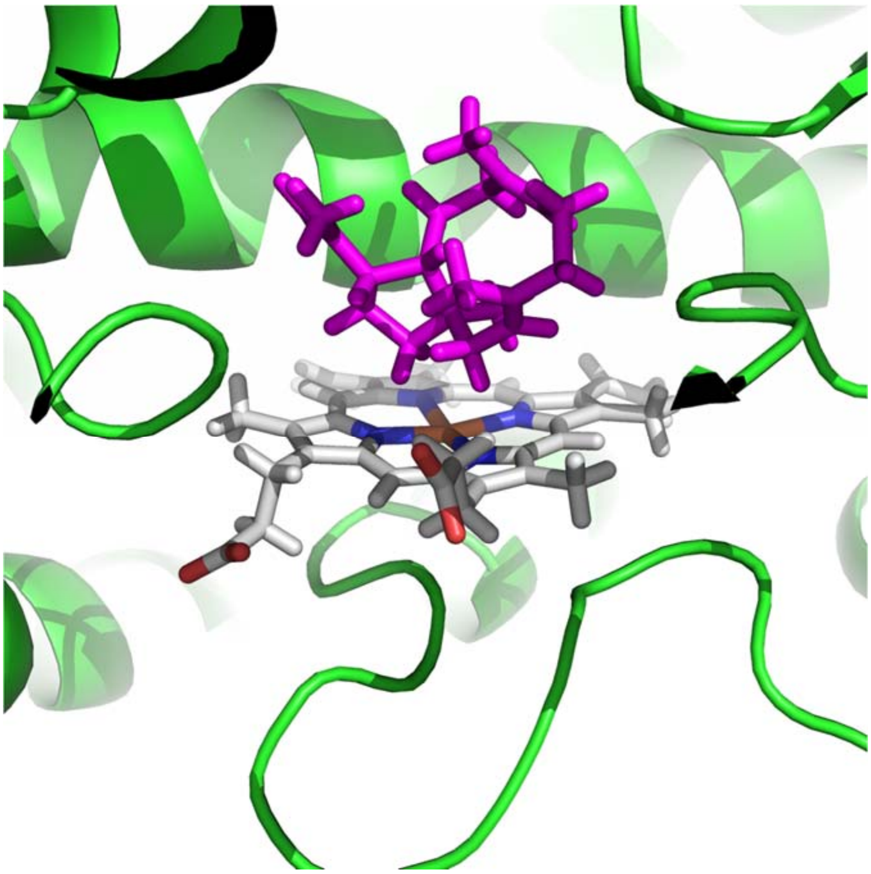

Supplementary Figure 13. Modelled $P450_{BM3}$ F87A (green) contains prosthetic heme group (gray) and docked (1R,3E,7E,11S,12S)-3,7,18-dolabellatriene (magenta). The heme group contains the iron cation (orange). (1R,3E,7E,11S,12S)-3,7,18-dolabellatriene is shown in the conformation comprising the lowest binding energy derived from cluster II.

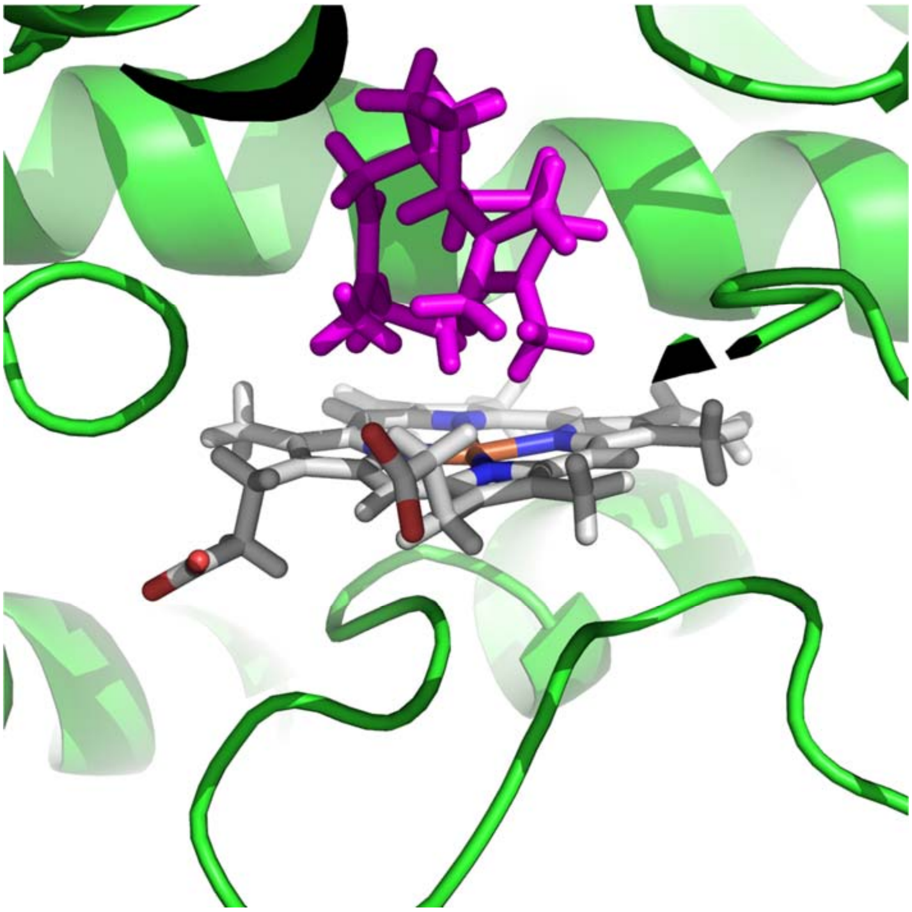

Supplementary Figure 14. Modelled P450$_{BM3}$ F87A/A328L (green) contains prosthetic heme group (gray) and docked (1R,3E,7E,11S,12S)-3,7,18-dolabellatriene (magenta). The heme group contains the iron cation (orange). (1R,3E,7E,11S,12S)-3,7,18-dolabellatriene is shown in the conformation comprising the lowest binding energy derived from cluster I.

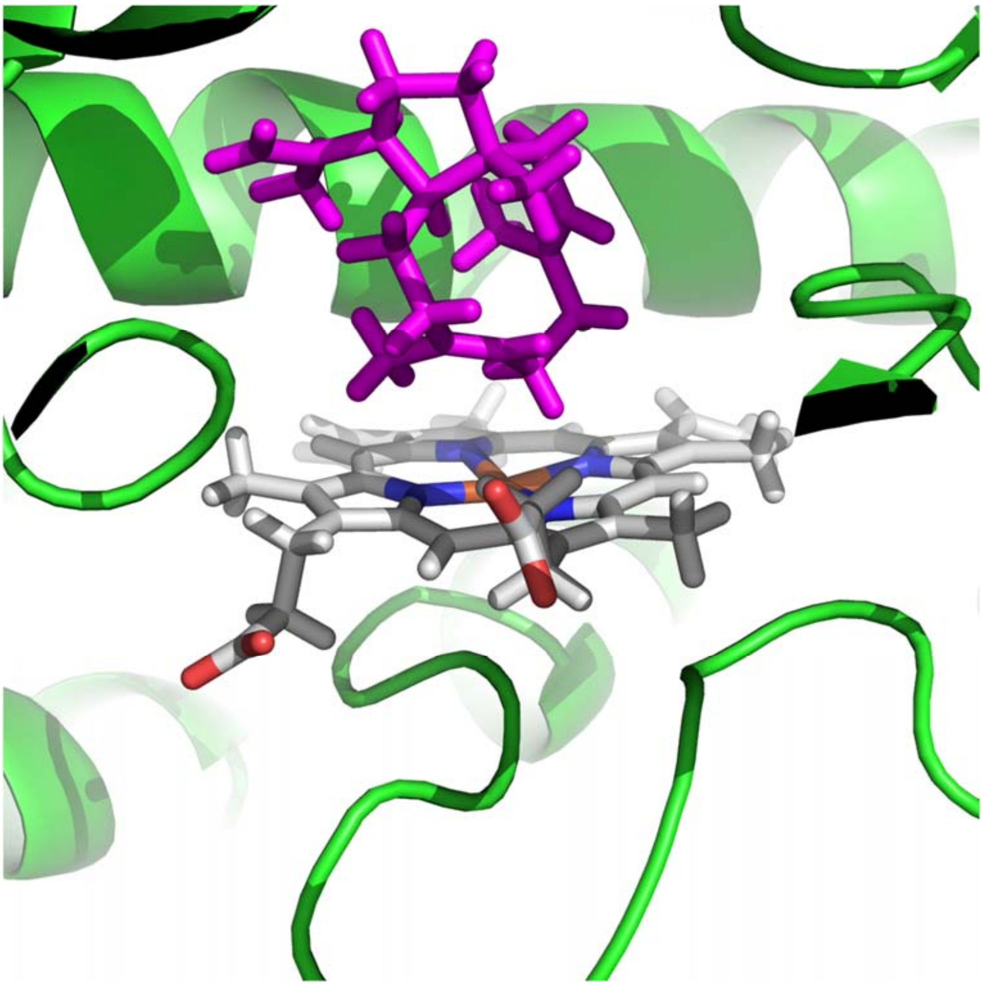

Supplementary Figure 15. Modelled P450$_{BM3}$ F87A/A328L (green) contains prosthetic heme group (gray) and docked (1R,3E,7E,11S,12S)-3,7,18-dolabellatriene (magenta). The heme group contains the iron cation (orange). (1R,3E,7E,11S,12S)-3,7,18-dolabellatriene is shown in the conformation comprising the lowest binding energy derived from cluster II.

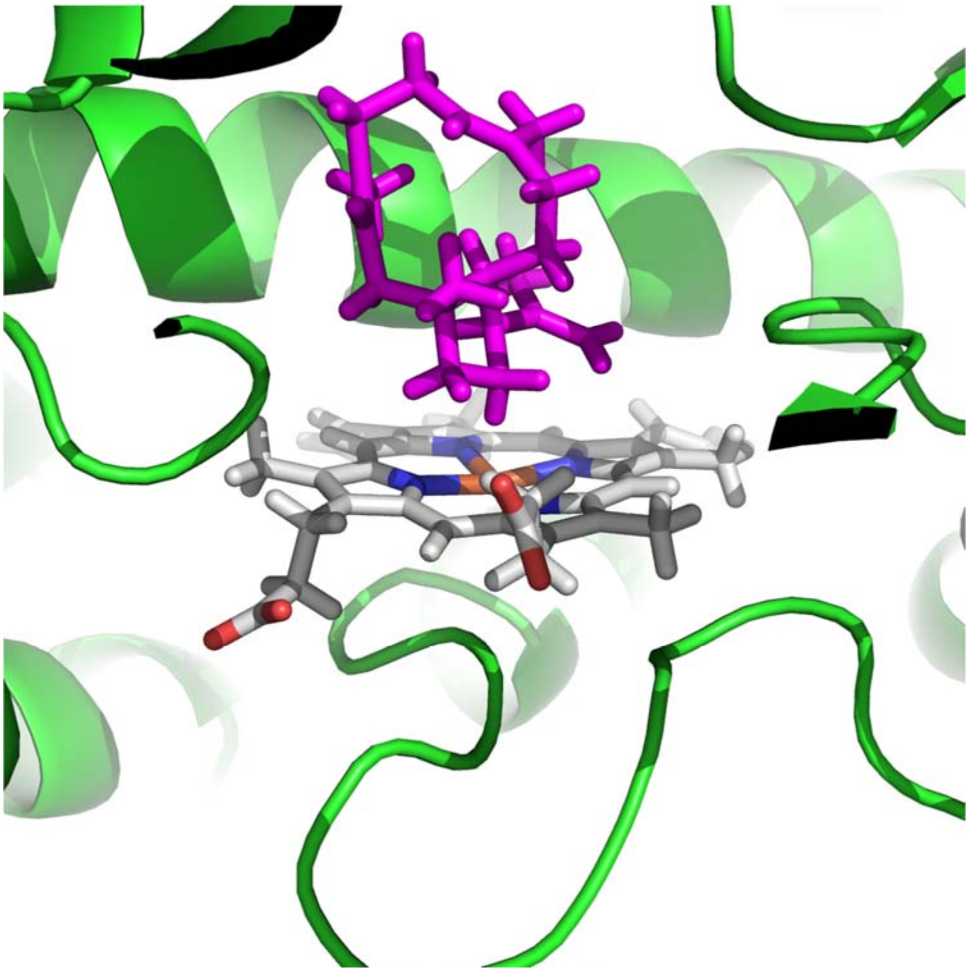

4. Genes

Supplementary Figure 13. Gene of 1-deoxy-D-xylulose 5-phosphate synthase (*dxs*)

CCATGGATGAGTTTTGATATTGCCAAATACCCGACCCTGGCACTGGTCGACTCCACCCAG
GAGTTACGACTGTTGCCGAAAGAGAGTTTACCGAAACTCTGCGACGAACTGCGCCGCTA
TTTACTCGACAGCGTGAGCCGTTCCAGCGGGCACTTCGCCTCCGGGCTGGGCACGGTCG
AACTGACCGTGGCGCTGCACTATGTCTACAACACCCCGTTTGACCAATTGATTTGGGATG
TGGGGCATCAGGCTTATCCGCATAAAATTTTGACCGGACGCCGCGACAAAATCGGCACC
ATCCGTCAGAAAGGCGGCCTGCACCCGTTCCCGTGGCGCGGCGAAAGCGAATATGACGT
ATTAAGCGTCGGGCATTCATCAACCTCCATCAGTGCCGGAATTGGTATTGCGGTTGCTGC
CGAGAAAGAAGGCAAAAATCGCCGCACCGTCTGTGTCATTGGCGATGGCGCGATTACCG
CTGGCATGGCGTTTGAAGCGATGAATCACGCGGGCGATATCCGTCCTGATATGCTGGTG
GTCCTCAACGACAATGAAATGTCGATTTCCGAAAATGTCGGCGCGCTCAATAACCATCT
GGCACAGCTGCTTTCCGGTAAGCTTTACTCTTCGCTGCGCGAAGGCGGGAAAAAAGTTT
TCTCTGGCGTTCCGCCAATTAAAGAGCTGCTCAAACGTACCGAAGAACATATTAAAGGC
ATGGTAGTGCCTGGCACGTTGTTTGAAGAGCTGGGCTTTAACTACATCGGCCCGGTTGAC
GGTCACGATGTGCTGGGGCTTATCACCACGCTGAAGAACATGCGCGACCTGAAAGGCCC
GCAGTTCCTGCATATCATGACCAAAAAAGGTCGTGGTTATGAACCGGCAGAAAAAGACC
CCATCACTTTCCACGCCGTGCCTAAATTTGATCCCTCCAGCGGTTGTTTGCCGAAAAGTA
GCGGCGGTTTGCCGAGCTATTCAAAAATCTTTGGCGACTGGTTGTGCGAAACGGCAGCG
AAAGACAACAAGCTGATGGCGATTACTCCGGCGATGCGTGAAGGTTCCGGCATGGTCGA
GTTTTCACGTAAATTCCCGGATCGTTACTTCGACGTGGCAATCGCCGAGCAACACGCGGT
GACCTTTGCCGCCGGTCTGGCGATTGGTGGGTACAAACCCATTGTCGCGATTTACTCCAC
TTTCCTGCAACGCGCCTATGATCAGGTGCTGCATGACGTGGCGATTCAAAAGCTCCCGGT
CCTGTTCGCCATCGACCGCGCGGGCATTGTTGGTGCTGACGGTCAAACCCATCAGGGCG
CTTTTGACCTCTCTTACCTGCGCTGTATACCGGAAATGGTCATTATGACCCCGAGCGATG
AAAACGAATGTCGCCAGATGCTCTATACCGGCTATCACTATAACGACGGCCCGTCCGCG
GTGCGCTACCCGCGCGGTAACGCGGTTGGCGTGGAACTGACGCCGCTGGAAAAACTGCC
AATTGGCAAAGGCATTGTGAAGCGTCGTGGCGAGAAACTGGCGATCCTTAACTTTGGTA
CGCTGATGCCAGACGCGGCGAAAGTCGCTGAATCGCTGAACGCTACGCTGGTCGATATG
CGTTTTGTGAAACCGCTTGATGAAGCGTTAATTCTGGAAATGGCCGCCAGCCATGAAGC
GCTGGTCACCGTAGAAGAAAACGCCATTATGGGCGGCGCAGGCAGCGGCGTGAACGAA
GTGCTAATGGCCCATCGTAAACCAGTACCCGTGCTGAACATTGGCCTGCCTGACTTCTTT
ATTCCACAAGGAACTCAGGAAGAAATGCGCGCCGAACTCGGCCTCGATGCCGCCGGTAT
GGAAGCCAAAATCAAGGCCTGGCTGGCATAAGAATTC

Supplementary Figure 14. Gene of 1-deoxy-D-xylulose 5-phosphate reductoisomerase (*dxr*)

CATATGAAGCAACTCACCATTCTGGGCTCGACCGGCTCGATTGGTTGCAGCACGCTGGA
CGTGGTGCGCCATAATCCCGAACACTTCCGCGTAGTTGCGCTGGTGGCAGGCAAAAATG
TCACTCGCATGGTAGAACAGTGCCTGGAATTCTCTCCCCGCTATGCCGTAATGGACGATG
AAGCGAGTGCGAAACTTCTTAAAACGATGCTACAGCAACAGGGTAGCCGCACCGAAGT
CTTAAGTGGGCAACAAGCCGCTTGCGATATGGCAGCGCTTGAGGATGTTGATCAGGTGA
TGGCAGCCATTGTTGGCGCTGCTGGGCTGTTACCTACGCTTGCTGCGATCCGCGCGGGTA
AAACCATTTTGCTGGCCAATAAAGAATCACTGGTTACCTGCGGACGTCTGTTTATGGACG
CCGTAAAGCAGAGCAAAGCGCAATTGTTACCGGTCGATAGCGAACATAACGCCATTTTT
CAGAGTTTACCGCAACCTATCCAGCATAATCTGGGATACGCTGACCTTGAGCAAAATGG
CGTGGTGTCCATTTTACTTACCGGGTCTGGTGGCCCTTTCCGTGAGACGCCATTGCGCGA
TTTGGCAACAATGACGCCGGATCAAGCCTGCCGTCATCCGAACTGGTCGATGGGGCGTA
AAATTTCTGTCGATTCGGCTACCATGATGAACAAAGGTCTGGAATACATTGAAGCGCGT
TGGCTGTTTAACGCCAGCGCCAGCCAGATGGAAGTGCTGATTCACCCGCAGTCAGTGAT
TCACTCAATGGTGCGCTATCAGGACGGCAGTGTTCTGGCGCAGCTGGGGGAACCGGATA
TGCGTACGCCAATTGCCCACACCATGGCATGGCCGAATCGCGTGAACTCTGGCGTGAAG
CCGCTCGATTTTTGCAAACTAAGTGCGTTGACATTTGCCGCACCGGATTATGATCGTTAT
CCATGCCTGAAACTGGCGATGGAGGCGTTCGAACAAGGCCAGGCAGCGACGACAGCAT
TGAATGCCGCAAACGAAATCACCGTTGCTGCTTTTCTTGCGCAACAAATCCGCTTTACGG
ATATCGCTGCGTTGAATTTATCCGTACTGGAAAAAATGGATATGCGCGAACCACAATGT
GTGGACGATGTGTTATCTGTTGATGCGAACGCGCGTGAAGTCGCCAGAAAAGAGGTGAT
GCGTCTCGCAAGCTGACTCGAG

Supplementary Figure 15. Bi-cistronic operon of 2-C-methyl-D-erythriol 4-phosphate cytidyltransferase synthase (*ispD*) 2-C-methyl-D-erythritol 2,4-cyclodiphosphate synthase (*ispF*)

<u>CCATGG</u>ATGGCAACCACTCATTTGGATGTTTGCGCCGTGGTTCCGGCGGCCGGATTTGGC
CGTCGAATGCAAACGGAATGTCCTAAGCAATATCTCTCAATCGGTAATCAAACCATTCTT
GAACACTCGGTGCATGCGCTGCTGGCGCATCCCCGGGTGAAACGTGTCGTCATTGCCAT
AAGTCCTGGCGATAGCCGTTTTGCACAACTTCCTCTGGCGAATCATCCGCAAATCACCGT
TGTAGATGGCGGTGATGAGCGTGCCGATTCCGTGCTGGCAGGTTTGAAAGCCGCTGGCG
ACGCGCAGTGGGTATTGGTGCATGACGCCGCTCGTCCTTGTCTGCATCAGGATGACCTCG
CGCGATTGTTGGCGTTGAGCGAAACCAGCCGCACGGGAGGGATCCTAGCCGCACCAGTG
CGCGATACGATGAAACGTGCCGAACCGGGCAAAAATGCCATTGCTCATACCGTTGATCG
CAACGGCTTATGGCACGCGCTGACGCCGCAATTTTTCCCTCGTGAGCTGTTACATGACTG
TCTGACGCGCGCTCTAAATGAAGGCGCGACTATTACCGACGAAGCCTCGGCGCTGGAAT
ATTGCGGATTCCATCCTCAGTTGGTCGAAGGCCGTGCGGATAACATTAAAGTCACGCGC
CCGGAAGATTTGGCACTGGCCGAGTTTTACCTCACCCGAACCATCCATCAGGAGAATAC
ATAAGCAGGAGCAGGAGCAGAAGGAGGAGCAGGAATGCGAATTGGACACGGTTTTGAC
GTACATGCCTTTGGCGGTGAAGGCCCAATTATCATTGGTGGCGTACGCATTCCTTACGAA
AAAGGATTGCTGGCGCATTCTGATGGCGACGTGGCGCTCCATGCGTTGACCGATGCATT
GCTTGGCGCGGCGGCGCTGGGGGATATCGGCAAGCTGTTCCCGGATACCGATCCGGCAT
TTAAAGGTGCCGACAGCCGCGAGCTGCTACGCGAAGCCTGGCGTCGTATTCAGGCGAAG
GGTTATACCCTGGGCAACGTCGATGTCACTATCATCGCTCAGGCACCGAAGATGTTGCC
GCACATTCCACAAATGCGCGTATTTATTGCCGAAGATCTCGGCTGCCATATGGATGATGT
TAACGTGAAAGCCACTACTACGGAAAAACTTGGATTTACCGGACGTGGGGAAGGGATTG
CCTGTGAAGCGGTGGCGCTACTCATTAAGGCAACAAAATGA<u>GAATTC</u>

Supplementary Figure 16. Gene of Isopentenyl-diphosphate delta isomerase (*idi*)

<u>CATATG</u>CAAACGGAACACGTCATTTTATTGAATGCACAGGGAGTTCCCACGGGTACGCT
GGAAAAGTATGCCGCACACACGGCAGACACCCGCTTACATCTCGCGTTCTCCAGTTGGC
TGTTTAATGCCAAAGGACAATTATTAGTTACCCGCCGCGCACTGAGCAAAAAAGCATGG
CCTGGCGTGTGGACTAACTCGGTTTGTGGGCACCCACAACTGGGAGAAAGCAACGAAGA
CGCAGTGATCCGCCGTTGCCGTTATGAGCTTGGCGTGGAAATTACGCCTCCTGAATCTAT
CTATCCTGACTTTCGCTACCGCGCCACCGATCCGAGTGGCATTGTGGAAAATGAAGTGT
GTCCGGTATTTGCCGCACGCACCACTAGTGCGTTACAGATCAATGATGATGAAGTGATG
GATTATCAATGGTGTGATTTAGCAGATGTATTACACGGTATTGATGCCACGCCGTGGGC
GTTCAGTCCGTGGATGGTGATGCAGGCGACAAATCGCGAAGCCAGAAAACGATTATCTG
CATTTACCCAGCTTAAATAA<u>CTCGAG</u>

Supplementary Figure 17. Gene of *cotB2*

<u>CATATG</u>ACCACCGGTCTGAGCACCGCAGGCGCACAGGATATTGGTCGTAGCAGCGTTCG
TCCGTATCTGGAAGAATGTACCCGTCGTTTTCAAGAAATGTTTGATCGTCATGTTGTTAC
CCGTCCGACCAAAGTTGAACTGACCGATGCAGAACTGCGTGAAGTTATTGATGATTGTA
ATGCAGCAGTTGCACCGCTGGGTAAAACCGTTAGTGATGAACGTTGGATTAGCTATGTT
GGTGTTGTTCTGTGGTCACAGAGTCCGCGTCATATTAAAGATATGGAAGCATTTAAAGC
CGTGTGCGTTCTGAATTGTGTTACCTTTGTTTGGGATGATATGGACCCTGCACTGCATGA
TTTTGGTCTGTTTCTGCCTCAGCTGCGTAAAATTTGCGAAAAATACTATGGTCCGGAAGA
TGCCGAAGTTGCCTATGAAGCAGCACGTGCATTTGTTACCAGCGATCACATGTTTCGTGA
TAGCCCGATTAAAGCAGCACTGTGTACCACCAGTCCGGAACAGTATTTTCGTTTTCGTGT
TACCGATATTGGCGTGGATTTTTGGATGAAAATGAGCTATCCGATTTATCGCCATCCGGA
ATTTACCGAACATGCAAAAACCAGCCTGGCAGCACGTATGACCACCCGTGGTCTGACCA
TTGTTAATGATTTCTATAGCTATGATCGCGAAGTTAGCCTGGGTCAGATTACCAATTGTT
TTCGTCTGTGTGATGTGAGTGATGAAACCGCCTTTAAAGAATTTTTCAGGCACGTCTGG
ATGACATGATCGAAGATATTGAATGCATCAAAGCGTTTGATCAGCTGACACAGGATGTT
TTTCTGGATCTGATTTATGGCAATTTTGTGTGGACCACCTCCAACAAACGTTATAAAACC
GCAGTGAATGATGTGAACAGCCGTATTCAATAA<u>CTCGAG</u>
